# Octopus-Inspired Underwater Gripper with Rapid Stiffness Tuning and Robot Enabling Upward Transport

**DOI:** 10.34133/cbsystems.0528

**Published:** 2026-03-31

**Authors:** Mingxin Wu, Yurong Liu, Jiaxi Wu, Waqar Hussain Afridi, Xingwen Zheng, Chen Wang, Guangming Xie

**Affiliations:** ^1^ National Center for International Joint Research of Micro-Nano Molding Technology, School of Mechanics and Safety Engineering, Zhengzhou University, Zhengzhou 450001, China.; ^2^ State Key Laboratory for Turbulence and Complex Systems, Intelligent Biomimetic Design Lab, School of Advanced Manufacturing and Robotics, Peking University, Beijing 100871, China.; ^3^ Department of Mechanical Engineering, National University of Singapore, Singapore 117575, Singapore.; ^4^ Institute of Cyber-Systems and Control, Department of Control Science and Engineering, Zhejiang University, Hangzhou 310027, China.; ^5^ Institute of Ocean Research, Peking University, Beijing 100871, China.

## Abstract

Underwater operations—such as marine environmental protection, resource recovery, and seabed exploration—require grippers with high adaptability. Existing rigid and soft grippers are constrained by their inherent material limitations, restricting their manipulation versatility. In this work, we introduce an octopus-inspired underwater gripper with rapidly tunable stiffness, integrated into an upward transport robot designed for efficient underwater object manipulation. Achieving softening in 1.3 s and rigidification in 0.8 s, the gripper demonstrates the shortest stiffness transition time reported to date, substantially advancing rapid and adaptive underwater manipulation. Emulating the octopus’s multimodal grasping strategy, the system can handle a wide range of objects—from light to heavy and soft to rigid—even in cluttered underwater environments. The integrated robot combines active buoyancy control with manipulation to enable continuous grasping and vertical transport of submerged objects. This study offers a robust solution for adaptive underwater manipulation, with potential applications in autonomous marine operations, ecological restoration, and ocean missions.

## Introduction

The ocean, covering over 70% of Earth’s surface, is one of the planet’s most vital natural resources, yet research indicates that less than 5% of its expanse has been thoroughly explored. This limited understanding not only constrains efforts to protect marine ecosystems and harness oceanic resources but also poses substantial technical challenges for underwater operations, such as environmental protection, resource recovery, and scientific exploration [1,2]. Consequently, the development of advanced robotic technologies is essential to address these demands. There is an urgent need to design wide-range adaptive robotic grippers and anti-gravity robots capable of performing diverse underwater manipulation tasks.

In biological systems, organisms like plants [3] and animals [4] often demonstrate extraordinary abilities to manipulate objects by changing shape and adjusting stiffness in response to dynamic environmental conditions. Soft robots are primarily inspired by these biological systems to achieve high levels of flexibility and environmental adaptability [5,6]. Unlike rigid robots, soft robots are made of soft or stretchable materials. They have attracted great attention due to their excellent ability to adapt to complex environments and interact safely with surroundings [7]. Soft robots have found applications in various fields, including grippers [8–10], crawling [11–13] and swimming robots [14–17], and minimally invasive surgical devices [18]. However, the inherently low stiffness of materials, such as silicone elastomers, makes soft robotic grippers perform poorly in tasks requiring high load capacities, like grasping and manipulating heavy objects. To address this shortcoming, various variable stiffness mechanisms have been proposed [19,20], including shape memory polymers (SMPs) [21–23], electrorheological materials [24], low-melting-point alloys [25], particulate or layered jamming structures [26,27], and elastomers filled with electro/magnetically active fluids [28,29]. Heat-activated SMPs have garnered considerable attention among these solutions due to their exceptional stiffness variability. However, they still encounter challenges such as slow response speed and limited deformation, which hinder efficient grasping and manipulation of complex, irregularly shaped underwater debris in dynamic environments. More importantly, a key challenge lies in studying how to achieve rapid stiffness control for soft robotic grippers in underwater environments.

Working underwater for a long time can cause severe damage to divers’ bodies and minds [30]. Underwater tasks such as cleaning up solid pollutants, resource recovery, and scientific research are highly labor-intensive; underwater robots have become essential tools that can even fully replace human labor in carrying out these missions [31]. However, to meet this demand, not only must underwater robots be able to manipulate multiple types of objects adaptively but also their operations must be gentle and noiseless to ensure that they are safe and do not cause harmful interference to the underwater ecosystem [32]. At present, the design of underwater robots can be divided into 2 main categories: zoomorphic and uncrewed vehicle types (remotely operated vehicles or autonomous underwater vehicles). However, existing uncrewed vehicle types typically use propellers or water jet-based propulsion systems [33], which can generate noise and vibration during outdoor field operations. The appearance of these vehicles, which are usually large and rigid like submarines, does not blend well with the marine environment [34]. Biomimicry potentially increases the ability of robots to get closer to marine life without disturbing them or the natural environment [35–37]. The octopus’s deformable soft body structure enables it to adapt and move freely in unstructured environments [38]. These characteristics make the octopus one of the popular animal models for guiding the design of a new generation of underwater robots. Developing a bionic octopus-like robotic gripper with the adaptive grasping ability to achieve the upward transportation of underwater objects has great application value.

**Fig. 1.**
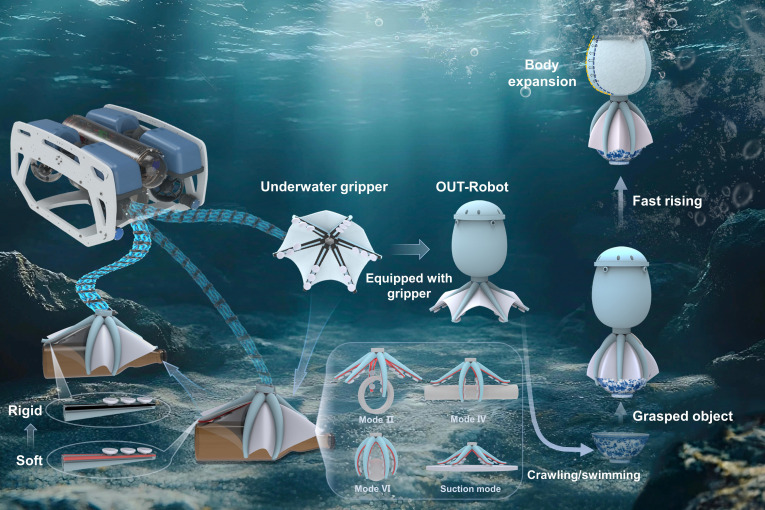
Octopus-inspired gripper with rapid variable stiffness and OUT-Robot. The gripper features 6 shape memory polymer (SMP)-embedded arms capable of rapid underwater stiffness tuning. In the soft state, it can perform suction mode or grasping mode, where positive pressure drives the arms before rapid stiffening locks the grasp. Independent arm control enables multiple grasping modes. The OUT-Robot comprises a soft outer layer and a rigid skeleton equipped with the gripper. Its internal drive system achieves jet propulsion swimming and arm-actuated underwater crawling. By adjusting internal shell pressure to control vertical buoyancy, the robot executes a continuous grasping operation cycle.

This work proposes a soft robotic gripper for underwater rapid stiffness variation and introduces an octopus-inspired upward transport robot (OUT-Robot) (Fig. 1). First, we propose a thermodynamic structural design that overcomes the thermal hysteresis bottleneck of underwater SMP actuators. By engineering a trilayer thermal interface that actively couples with the aquatic environment as a heat sink, we achieve rapid stiffness tuning. Second, we demonstrate a “Soft-Rigid Hybrid” manipulation strategy that decouples compliance from load capacity. By dynamically tuning stiffness during the grasping cycle, the system utilizes high compliance to facilitate sucker adhesion on irregular surfaces and high rigidity to lock the shape for heavy lifting, resolving the classic trade-off in soft robotics. Third, we establish a silent, buoyancy-driven “Pick-and-Float” operational paradigm. This mechanism utilizes phase-change shape locking and buoyancy control to separate energy-intensive grasping from energy-efficient transport, offering a sustainable solution for ecologically sensitive marine restoration.

## Materials and Methods

### Materials and equipment

The SMP utilized for the variable stiffness structures was polylactic acid (PLA). Soft elastomer components were cast using Dragon Skin 20 silicone. The electrothermal heating element consisted of a nickel–chromium alloy resistance wire. The robot’s control system was built around an Arduino Nano microcontroller.

### Fabrication and assembly

The complete OUT-Robot system comprises a variable stiffness gripper, a cast silicone soft shell, a 3-dimensional (3D)-printed internal skeleton, and a hydraulic drive system. The soft components were fabricated using a 3D-printing-assisted casting process. The variable stiffness arms were assembled by integrating a 3D-printed PLA core, wound with a heating wire, into a silicone actuator body with integrated suckers. The manufactured components were permanently assembled using silicone adhesives and compression sealing to ensure waterproofing. Detailed step-by-step fabrication and assembly procedures are provided in the Supplementary Materials.

### Experimental characterization

Mechanical performance and grasping forces were evaluated in an underwater environment (21 °C) using a motorized test stand equipped with a dynamometer. To accurately quantify the stiffness transition speed, a synchronized dual-measurement setup was employed, tracking both the SMP core temperature via an embedded thermocouple and the mechanical displacement under a standard load. Detailed testing protocols and specific parameters are available in the Supplementary Materials.

### Finite element analysis

Static nonlinear finite element analysis (FEA) was conducted using Abaqus/Standard to predict the arm’s bending angle, tip motion trajectory, and overall mechanical response. The simulation utilized 4-node linear tetrahedral elements (C3D4H). The material behavior was defined using the Yeoh hyperelastic model, with coefficients (*C*_10_ = 0.060,662,207,64, C_20_ = 0.002,238,763,41, and*C*_30_ = 1.853,197,581) derived from empirical mechanical property tests. These simulations guided prefabrication geometric optimization, such as selecting the inner wall thicknesses and establishing the optimal initial arm inclination angle (30°) to maximize the effective grasping workspace.

## Results

### Design and working principle of the arm

**Fig. 2.**
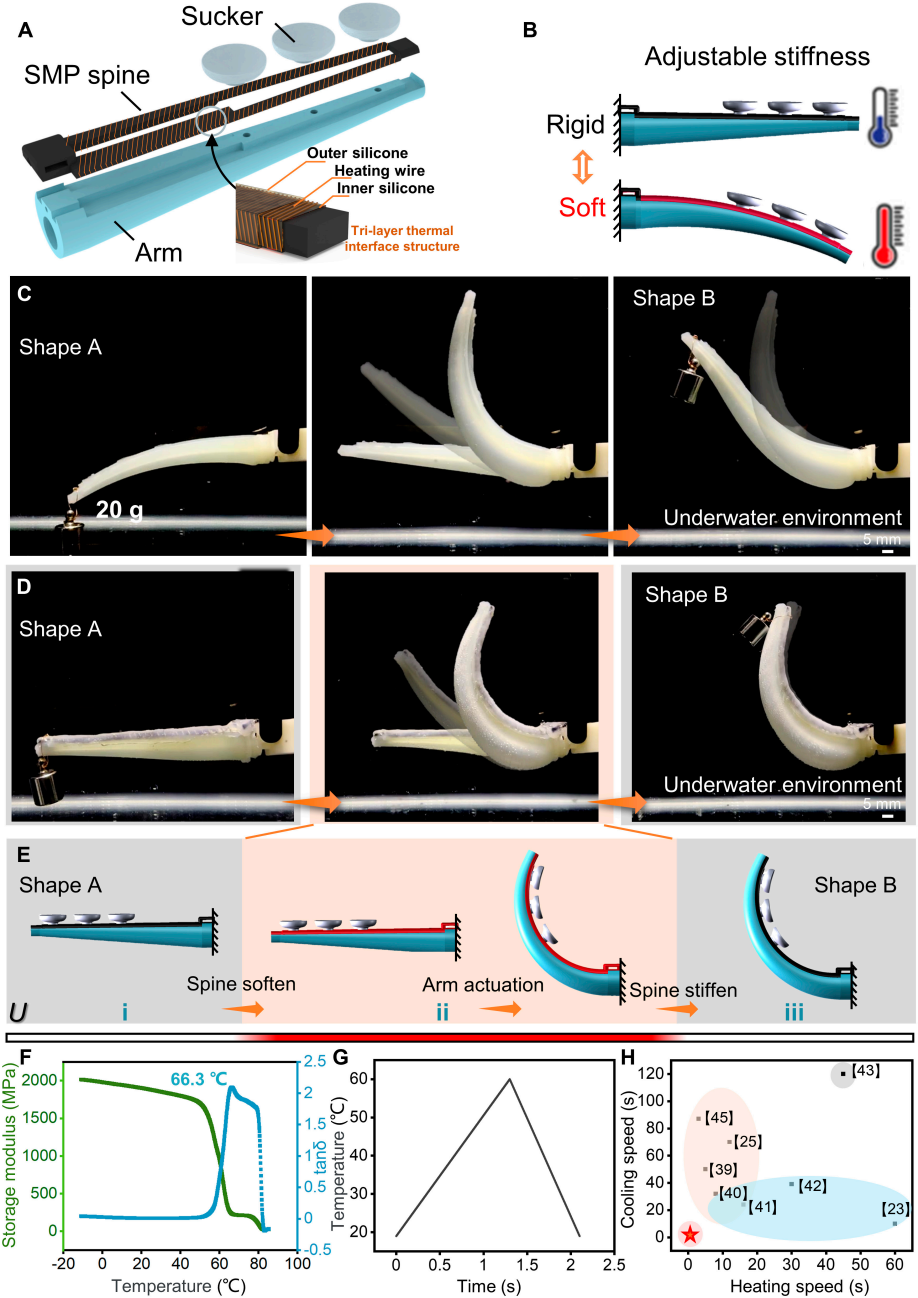
Structure and underwater performance of the SMP-equipped arm. (A) Single arm assembly with actuator, suckers, and variable stiffness system. (B) Mechanism of SMP switching between soft and rigid states via nickel–chromium wire heating/cooling. The shape locking is realized through SMP cooling, whose stiffness can be controlled by the Joule heating through a heating wire (U). (C and D) Comparison of load-bearing capacity of the arm (C) without SMP and (D) the SMP-equipped during the transition from shape A to shape B. (E) Shape-morphing processes of the SMP-equipped arm. (i) When U (voltage) is applied, the resistance wire heats the SMP, causing it to soften after the temperature exceeds its glass transition temperature *T*_g_. (ii) Once the SMP is fully flexible, pressure is applied to deform it. (iii) After the target shape is achieved, U is removed to allow the SMP to cool using the water temperature, thereby locking in the new shape. (F) The DMA characterization of PLA is chosen as the SMP material. (G) Measured heating and cooling times for stiffness variation underwater. (H) A comparison of stiffness variation speed with previous actuators shows significant improvement.

The structure of a single arm is shown in Fig. 2A, which is composed of an actuator, 3 suckers, and a variable stiffness system (see the “Fabrication and assembly” section in Materials and Methods; Figs. S1 and S2). The configuration of 6 arms was selected as an optimal trade-off to enable multiple symmetric grasping modes (modes II, IV, and VI) while minimizing the control system’s weight and complexity. Similarly, the arrangement of 3 suckers per arm was designed to maximize the effective suction area and provide adhesive redundancy on irregular surfaces, all within the spatial constraints of the flexible arm length. A dedicated through-channel for connecting the suckers in series is integrated inside the actuator to achieve coordinated control of the suckers. The sucker can generate suction via negative pressure to enhance the stability when grasping objects. Under positive pressure drive, the sucker can be actively separated from the object (Fig. S3 and Movie S1). We utilized SMP (PLA), chosen for its optimal glass transition temperature (*T*_g_ ≈ 66 °C). To achieve subsecond stiffness tuning, we engineered a specific trilayer thermal interface structure (inner silicone/heating wire/outer silicone), as shown in Fig. 2B and Fig. S1. This design creates a thermodynamic synergy among the material, geometry, and the underwater environment.

First, the inner silicone layer functions as a thermal diffuser. It transforms the high-power-density “line-source” heat from the nickel–chromium wire (0.2 mm diameter, 2 mm spacing) into a uniform “surface-source” heat flux. This homogenization prevents localized burning of the PLA while ensuring that the bulk material softens synchronously.

Second, the outer silicone encapsulation acts as a transient thermal barrier. Due to silicone’s low thermal conductivity, it temporarily impedes heat dissipation into the water during the short actuation phase (1.3 s), effectively confining thermal energy to the SMP core for rapid heating.

Finally, the rigidification process exploits the underwater environment as an active heat sink. The high convective heat transfer coefficient of water ($h_{\text{water}}\gg h_{\mathrm{air}}$) combined with the large temperature gradient (Δ*T*) overcomes the thermal resistance once heating stops. This structural optimization minimizes the system’s thermal time constant (*τ*) and keeps the Biot number low, enabling the rapid cooling (0.8 s) and stiffness locking that is unattainable in air-based systems.

The loadbearing capacity of an arm equipped with the SMP is compared with that of an arm without SMP (Fig. 2C and D and Movie S2). Initially, the arm functions as a rigid structure (straight “shape A”) to hold a mass of 20 g close to the free end (Fig. 2D), while the arm without SMP cannot (Fig. 2C). After removing the mass, the SMP is softened by applying a voltage of *U* = 36 V to the nickel–chromium wire wrapped around the SMP (Fig. 2E). As the SMP softens, it can bend freely to any angle when the arm is driven (Fig. 2D and E). After the module is bent to an angle of 100°, the voltage applied to the SMP is stopped (*U* = 0), and it rapidly cools and recovers its rigidity at water temperature (21 °C). The arm holds a benchmark mass of 20 g without additional energy input in the “shape B” rigid structure (visualized to demonstrate stiffness variation), while the arm (no SMP) end has a large deflection. The SMP enhances the rigidity of the end of the arm, which is beneficial for improving the load capacity of the gripper during grasping, thereby realizing the grasping of more types of objects.

To investigate the thermomechanical behavior of the SMP material, we performed a dynamic mechanical analysis (DMA) test on a DMA analyzer (Netzsch DMA242E, Germany) by decreasing the temperature from 90 to −30 °C at a rate of 2 °C/min. We identified the *T*_g_ of the SMP material to be around 66 °C according to the peak of tanδ (the ratio of the loss and storage moduli). Fig. 2A shows that the storage modulus (corresponding to elastic response) increases remarkably from ≈26 MPa at high temperatures (>70 °C) to more than 2 GPa at room temperature. This sharp transition corresponds to the vitrification of the polymer chains as the temperature drops below *T*_g_, restricting segmental motion and recovering the material’s high mechanical rigidity. This thermomechanical process is fully reversible, allowing the gripper to cycle between soft (rubbery) and rigid (glassy) states repeatedly. Next, the switching speed of the soft and rigid arms under various voltage conditions was measured in an underwater environment (21 °C) (Fig. S4).

To quantify the interplay between geometric parameters and stiffness tuning speed, we established a physics-informed model coupling Joule heating with transient heat transfer (detailed derivation in the Supplementary Materials). The transient temperature response *T*(*t*) of the arm is governed by:

$$T\left(t\right)=T_{0}+A\frac{I_{2}\;}{d_{2}}\left(1-e^{-t/\tau }\right)$$ (1)

where *T*_0_ is the ambient water temperature, *A* is the electrothermal coupling constant, *d* is the wire diameter, and *τ* is the thermal time constant determined by the system’s heat capacity and convective heat transfer coefficient ($h_{\text{water}}$). This model reveals that the heating rate scales with $I_{2}/d_{2}$, while the cooling/locking phase is dominated by *τ*. Based on this scaling law and experimental validation (Fig. S4A), we selected a wire diameter of d=0.2mm. This diameter offers an optimal trade-off: It reduces the thermal mass compared to thicker wires (lowering *τ* for faster switching) while maintaining sufficient structural integrity compared to thinner wires (0.1 to 0.15 mm) and requiring lower activation currents than thicker wires (0.25 to 0.3 mm). Coupling this thermal model with the PLA’s temperature-dependent modulus (Fig. 2F) allows for prediction of the stiffness evolution *H*(*I*,*d*,*t*).

As shown in Fig. 2G, when the voltage is set to 36 V while the current is 1.6 A, the time required for heating to soften is 1.3 s, and the time for cooling to rigidity is 0.8 s. The stiffness variable of the arm proposed in this work is compared with the stiffness variation speed of various actuators developed previously (Fig. 2H) [23,25,39–44], and the speed is substantially improved. In practical applications, the response time can be markedly reduced when grasping different objects, thereby improving work efficiency. While previous SMP-based pneumatic actuators typically require actuation pressures of 150 to 200 kPa to achieve deformation [23,45], our optimized silicone structure achieves full bending at a lower pressure of 100 kPa. In terms of load capacity, the cooperative grasping force of our gripper (>4 N, mode VI) is competitive with existing soft-rigid fingers (typically 1 to 3 N [42,45]). Most notably, as shown in Fig. 2H, our design leverages the underwater environment to reduce the stiffness transition time to ~1 s, overcoming the primary disadvantage of terrestrial SMP actuators—slow cooling rates (>30 s) [25,43,45]. Furthermore, unlike hybrid jamming grippers that require continuous vacuum power [42], our SMP strategy offers the advantage of “zero-energy” shape locking.

### Performance characterization of the single arm unit

**Fig. 3.**
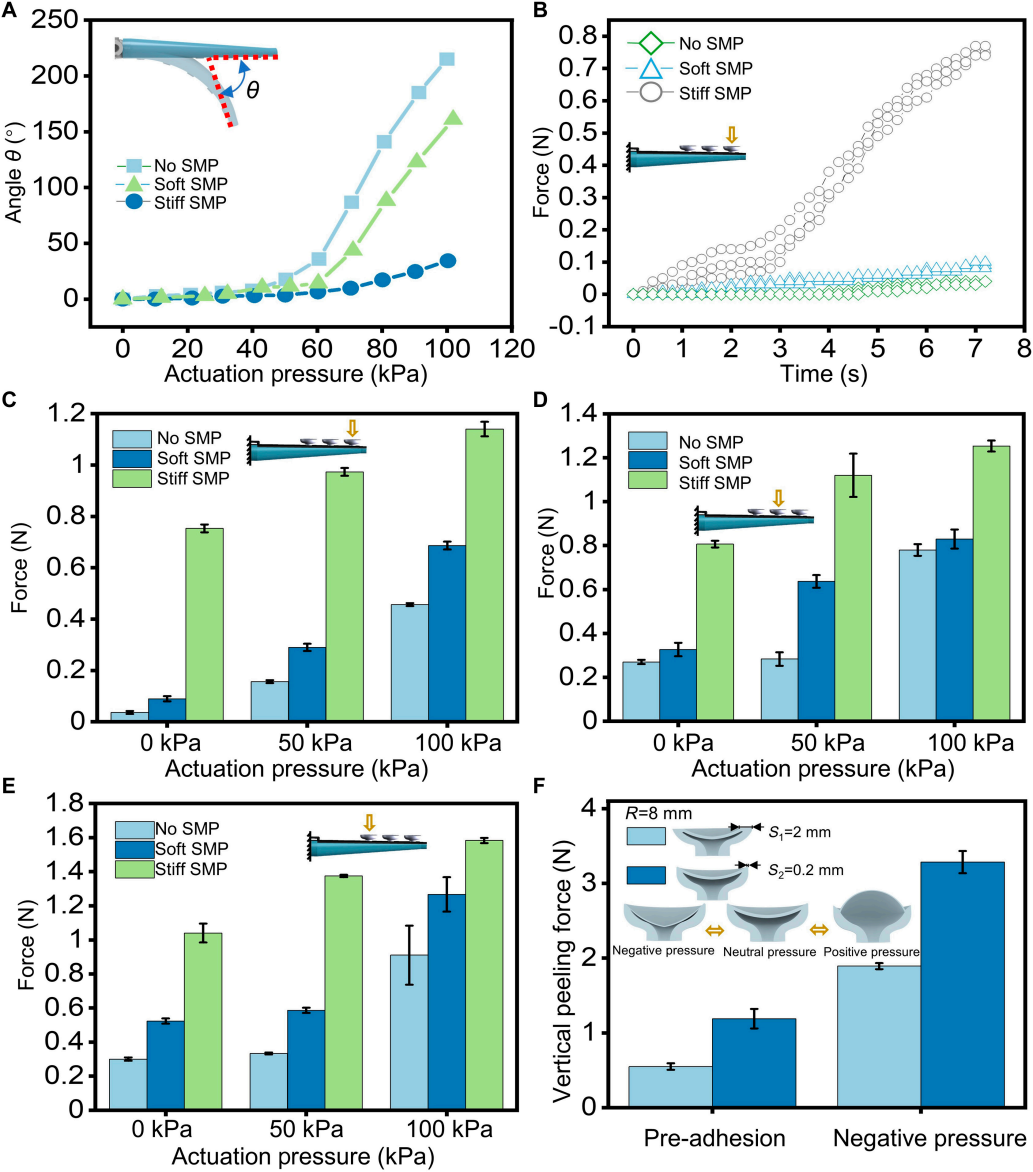
Performance comparison and characteristics of multiple types of arms. (A) Bending angle comparison among No SMP, Soft SMP, and Stiff SMP. Stiff SMP substantially suppresses bending. (B) Real-time stiffness changes at the first sucker point on the arm at 0 kPa. No SMP and Soft SMP maintain low stiffness, while the Stiff SMP arm exhibits marked force changes, with stiffness about 25 times higher than that of the No SMP arm. (C to E) Stiffness measurements at 3 points on multiple types of arms under different driving pressures (0, 50, and 100 kPa). SMP substantially increases arm stiffness, especially at the tip (C), enhancing grasping stability. (F) Performance evaluation of a single sucker on the arm. The improved sucker increased pre-adhesion force by a factor of approximately 2.2 and suction force in the negative pressure state by a factor of about 1.7.

Fig. 3 shows the performance comparison among multiple types of arms: (a) arm without SMP (No SMP); (b) arm with SMP in a softened state (Soft SMP); and (c) arm with SMP in a rigid state (Stiff SMP). As shown in Fig. 3A, the bending angle *θ* of the Stiff SMP is only 34.38° at 100 kPa, while the bending angle of the Soft SMP is 161°. The SMP with variable stiffness substantially suppresses the bending of the arm. The adaptive bending ability of the Soft SMP arm was verified in an underwater environment, and the Soft SMP will form joints at the points of contact (Fig. S5 and Movie S2). The arm equipped with the SMP exhibits the high flexibility of a soft robot when softened and can achieve adaptive grasping of irregular objects.

The stiffness of the arm at 3 different points (sucker positions) along the arm was measured separately through a single arm performance characterization system (Fig. S6). As shown in Fig. 3B, the real-time change of the stiffness at the first point of the arm when there is no external pressure (0 kPa) shows that No SMP and Soft SMP maintain low stiffness. As the motion stroke of the dynamometer probe increases, the Stiff SMP arm shows marked force changes, and the stiffness is about 25 times greater than that of the No SMP arm. Therefore, when the SMP on the arm turns rigid, the stiffness of the arm increases substantially to ensure the stability of the grasping.

Next, the stiffness at 3 points of different arms under different driving pressures was measured, as shown in Fig. 3C to E. At the first point (Fig. 3C), the stiffness of the Stiff SMP increased by a factor of ~25 compared with the No SMP at 0 kPa pressure. When a pressure of 50 kPa was applied, the stiffness increased by a factor of 6.6. When a pressure of 100 kPa was applied, the stiffness increased by a factor of 2.58. At the second point (Fig. 3D) and the third point (Fig. 3E), the SMP substantially increased the stiffness of the arm. Specifically, under 100 kPa pressure, the maximum output forces at the 3 measured points reached approximately 1.09 N (~112 g), 1.25 N (~128 g), and 1.56 N (~160 g), respectively. While increasing the SMP thickness could further enhance load capacity, the current thin profile was prioritized for rapid thermal response. Based on the above test results, although the SMP increased the stiffness of multiple positions of the arm, the stiffness increase was most pronounced at the tip of the arm even though the cross-section is narrowest near the tip, which is conducive to greatly improving the stability of the grasping.

Finally, the performance of a single sucker located on the arm was measured (Fig. 3F). The sucker was peeled off vertically along the vertical direction of the dynamometer, and the pre-adhesion force of the 2 suckers and the negative pressure suction force when the sucker was driven by a negative pressure of 60 kPa were measured. Compared with the previously proposed sucker with a diameter of 8 mm, the improved sucker reduces the flat area of the sucker edge, the pre-adhesion force is increased by a factor of ~2.2, and the suction force in the negative pressure state is increased by a factor of ~1.7. The performance of the sucker has been greatly improved in both aspects, and the ability to detach from the object under positive pressure is maintained, which is beneficial to the grasping of heavier objects.

### Multi-modal grasping strategies and force analysis

**Fig. 4.**
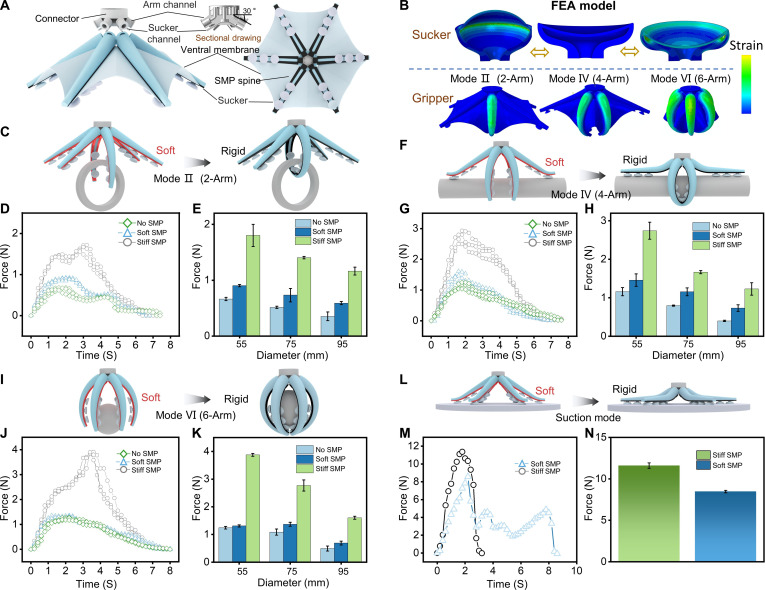
Variable stiffness gripper: structure, design verification, and performance evaluation. (A) The underwater gripper’s design is composed of a connector, 6 arms, and 6 ventral membranes. (B) Finite element analysis of the sucker and gripper. (C) Manipulation process of mode II. (D and E) Performance characterization of the gripper in mode II. (D) Real-time data changes for grasping an object with a 55-mm diameter. Dotted lines represent raw data from multiple experimental repetitions to demonstrate repeatability. (E) Comparison of grasping force among 3 different gripper designs, showing substantial improvement with Stiff SMP. (F) Manipulation process of mode IV. (G and H) Performance characterization of the gripper in mode IV. (G) Real-time data changes for grasping an object with a 55-mm diameter. (H) Comparison of grasping force, with Stiff SMP exhibiting more than twice the force of others across different diameters. (I) Manipulation process of mode VI. (J and K) Performance characterization of the gripper in mode VI. (J) Real-time data changes for grasping an object with a 55-mm diameter. (K) Comparison of grasping force, showing higher force in mode VI that increases with the number of arms. (L) Manipulation process of suction mode. (M and N) Performance characterization of the gripper in suction mode. (M) Real-time changes in suction force for 2 gripper designs. (N) Comparison of suction force magnitude between the 2 designs. Stiff SMP exhibits greater suction force, while Soft SMP enables redundant adhesion.

The structure of the gripper with variable stiffness is shown in Fig. 4A (see the “Fabrication and assembly” section in Materials and Methods; Figs. S7 and S8). The arm channel on the connector is designed with a 30° inclination to facilitate grasping with a lower pressure drive. The 6 arms’ channels on the connector independently control the bending of each arm, and the 6 channels for suckers in series are used to achieve reversible adhesion of the sucker. As shown in Fig. 4B, the design of the arm bending of the gripper and the feasibility of grasping modes II (2-Arm), IV (4-Arm), and VI (6-Arm) are verified by simulation (see the “Finite element analysis” section in Materials and Methods). In order to verify the effectiveness of stiffness variation, the SMP’s variable stiffness feature is combined with 6 arms to carry out grasping force test experiments (see Fig. S10 for the force measurement system). The pressure is set to 100 kPa for the test, the test process is driven by a slide rail with a dynamometer, and the stroke cycle starts from the grasping state to the object completely leaving the gripper’s hold. Three types of grippers were tested: (a) No SMP gripper; (b) Soft SMP gripper (where the arm remains in a softened state throughout the process); and (c) Stiff SMP gripper (where the arm becomes stiff after grasping is completed). The grasping of objects of 3 diameters was tested separately: The arm can wrap the object completely; the arm can wrap three-quarters of the grasped object; the arm can wrap half of the grasped object.

Mode II involves softening the arm first and then driving any 2 symmetrical arms to achieve grasping (Fig. 4C). Stiff SMP exhibits the largest grasping force and requires a greater force to detach the object. Soft SMP and No SMP have smaller grasping forces, and the object detachment trajectories are similar (Fig. 4D). The grasping force of the 3 grippers for objects of 3 different diameters was tested, and Stiff SMP substantially improved the gripping force compared to the other grippers (Fig. 4E). Mode IV involves softening the arm first and then driving any 4 symmetrical arms to achieve grasping (Fig. 4F). The grasping and detaching curves for Stiff SMP demonstrate a markedly greater grasping force (Fig. 4G). At different diameters, the grasping force of Stiff SMP is more than twice that of the other grippers (Fig. 4H). Mode VI softens the arms first and drives the 6 arms to bend to achieve grasping (Fig. 4I). Unlike modes II and IV, mode VI shows a higher grasping force exceeding 4 N (equivalent to >400 g payload capacity) (Fig. 4J). It is evident that as the number of arms increases, the resistance of the rigid arms that must be overcome when detaching the object also increases, leading to a higher grasping force. The improvement in stiffness provided by Stiff SMP is substantial, being 3 times that of No SMP.

In the suction grasping mode, the arms with an initial tilt angle can adaptively fit the object’s surface after softening (Fig. S9) and can grasp a variety of curved or flat objects (Fig. 4L). The suction force of the Stiff SMP gripper and the soft SMP gripper was tested under a −60 kPa drive. During the measurement, a smooth disk was attached to the sucker, and as the disk moved upward at a constant speed, the changes in the dynamometer readings were recorded in real time. Fig. 4M and N shows that the stiff SMP gripper exhibits a greater suction force in the suction grasping mode. However, the real-time change of suction force shows that the stiff SMP gripper will rapidly detach from the disk as the disk moves away, resulting in a sudden drop in suction force. For the soft SMP gripper, due to the compliance of the arm, the suckers on the arm detach in batches to achieve redundant adhesion. The gripper can precisely manipulate various objects by integrating stiffness variation and multiple grasping modes (Fig. S11).

### Multimodal grasping performance in underwater cluttered scenes with rapid stiffness variation

**Fig. 5.**
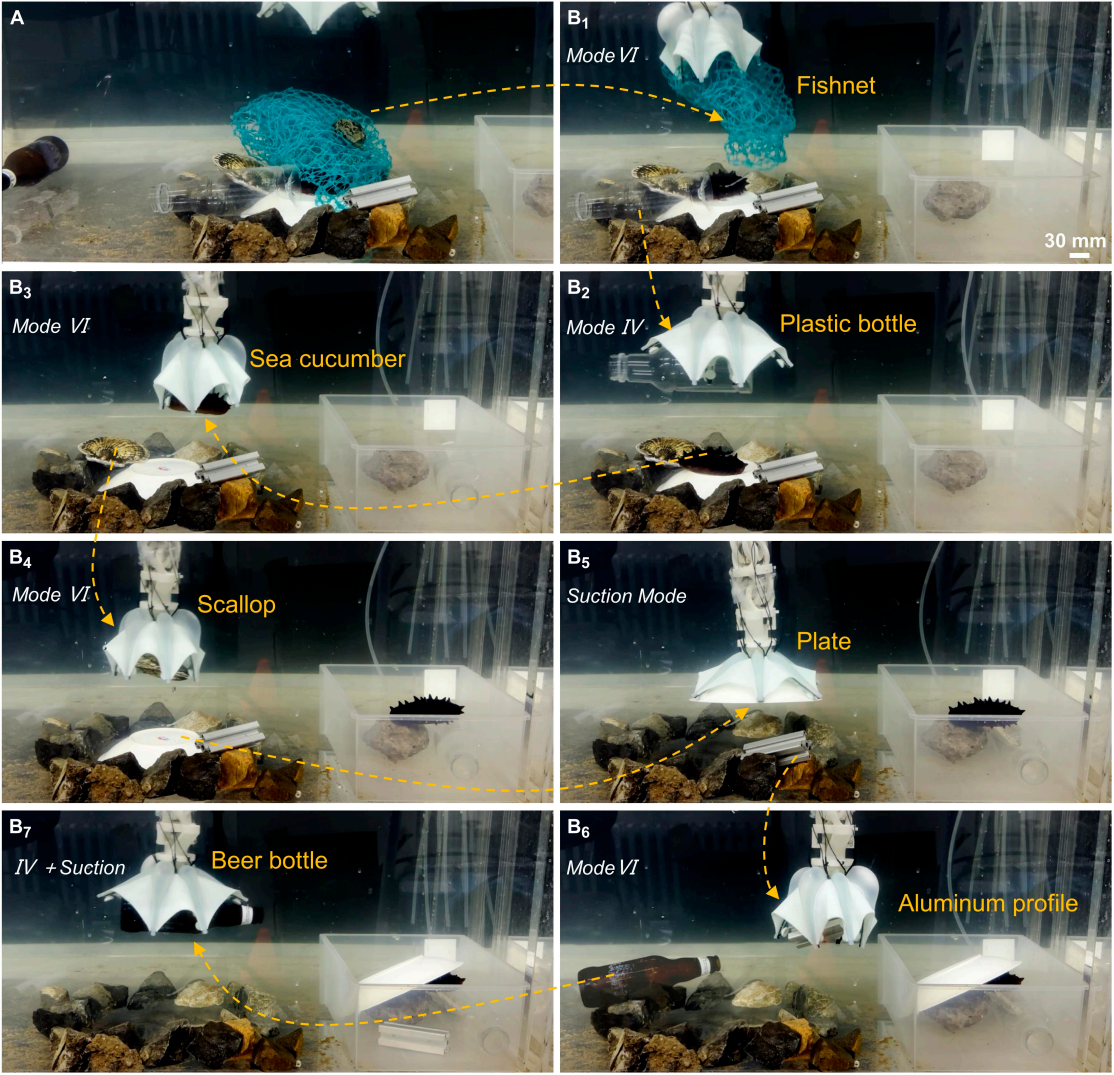
Demonstration of the gripper’s integrated multimodal grasping and rapid stiffness variation capabilities in a cluttered underwater environment. (A) Complex arrangement of heavy objects (beer bottle, aluminum profile, plate), biological resources (sea cucumber, scallop), and underwater pollutants (fishing net, plastic bottle) stacked underwater with stones as interference. (B_1_ to B_7_) Sequential manipulation by the gripper: (B_1_) mode VI for removing the fishing net; (B_2_) mode IV for grasping and transferring the plastic bottle; (B_3–4_) mode VI for collecting sea cucumbers and scallops; (B_5_) suction mode for nondestructively grasping the fragile plate; (B_6_) mode VI for grasping and transferring the aluminum profile; (B_7_) mode IV with suction mode assistance for grasping and transferring a heavy beer bottle. This sequence highlights the gripper’s ability to switch between grasping modes rapidly and vary stiffness, enabling effective manipulation of diverse and heavy objects in complex underwater environments.

A cluttered scene, as depicted in Fig. 5A, where multiple types of objects are stacked, demonstrates the benefits of the gripper’s integrated multimodal grasping and rapid stiffness variation grasping capabilities (Movie S3). Experiments were conducted in a laboratory pool at a depth of 2 m to validate the effectiveness of the grasp. The scene includes heavier objects (beer bottles, aluminum profiles, and plates), biological resources (sea cucumbers, scallops), and common underwater solid pollutants (abandoned fishing nets, plastic bottles). The above items are stacked underwater, surrounded by stones as interference. The plate is placed at the bottom, with scallops, sea cucumbers, aluminum profiles, and plastic bottles stacked on top in sequence. A layer of fishing net is laid on the top and pressed down with stones. First, the gripper switches to mode VI to remove the fishing net (approximately 0.9 g) above the objects (as shown in Fig. 5B_1_). Subsequently, it switches to mode IV to grasp and transfer the plastic bottle (Fig. 5B_2_). Next, the gripper switches to mode VI again to collect sea cucumbers and scallops (Fig. 5B_3–4_). After that, it switches to suction mode to nondestructively grasp the fragile plate (Fig. 5B_5_). Next, it switches to mode VI to grasp and transfer the aluminum profile (Fig. 5B_6_). Finally, it switches to mode IV and starts the suction mode for assistance, successfully grasping and transferring a heavy beer bottle (approximately 500 g) with a smooth surface (Fig. 5B_7_). In this complex underwater environment, the gripper showcased the advantage of rapid stiffness variation to boost the grasping force, successfully manipulating various heavy underwater objects. This demonstrates the system’s wide dynamic range, capable of manipulating objects from extremely light debris (<1 g) to heavy solid waste (>500 g). At the same time, the gripper can rapidly switch between multiple grasping modes when facing complex and diverse objects, achieving the grasping of multiple types of objects. This versatility highlights the application potential of this multimodal gripper with variable stiffness in handling complex objects within underwater unstructured environments.

### Design and application of the OUT-Robot

**Fig. 6.**
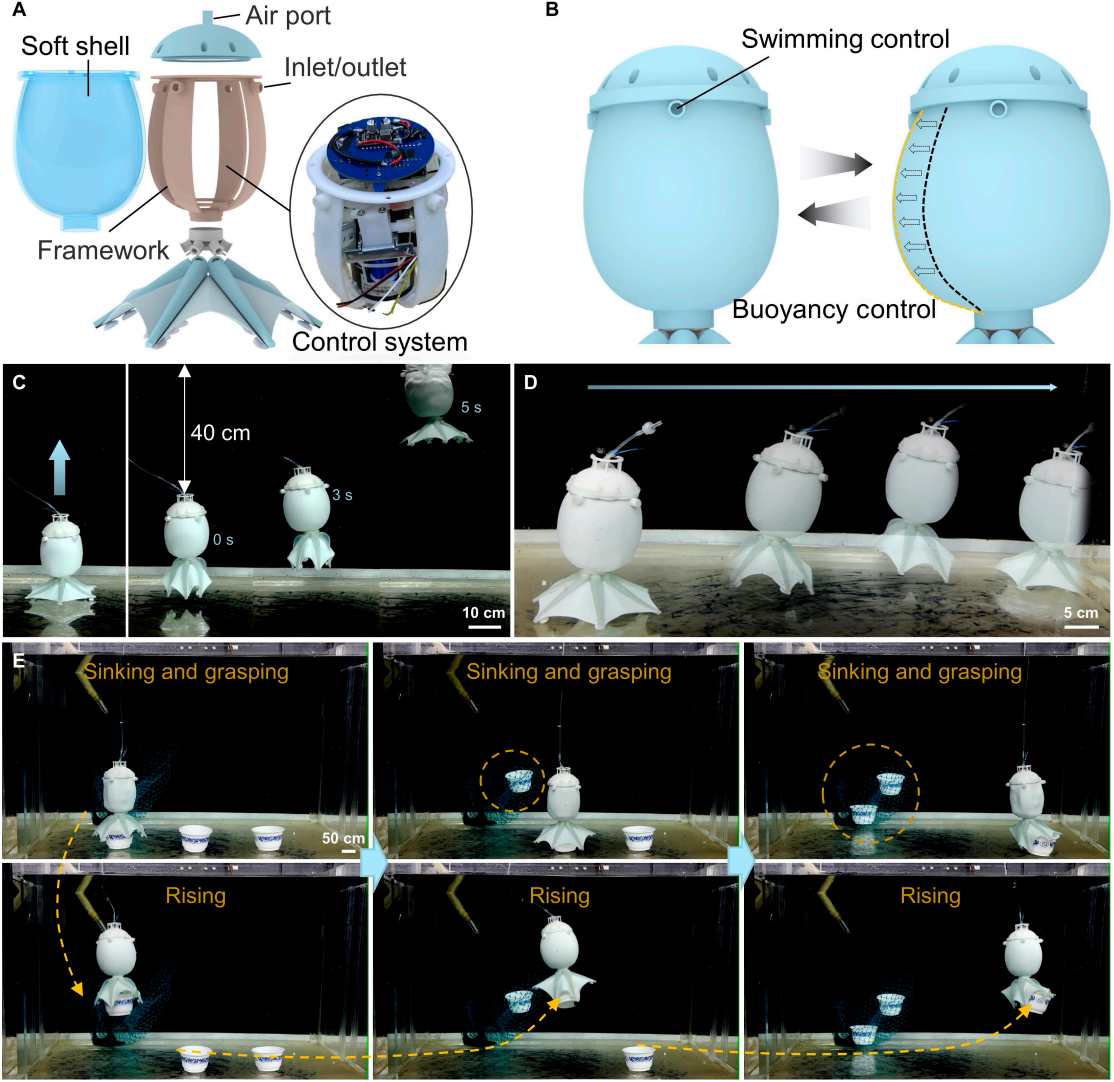
Design and performance evaluation of the OUT-Robot with variable stiffness gripper. (A) Schematic diagram depicting the robot’s design includes an external silicone soft shell, an internal skeleton, and a circuit board for controlling the water pump and solenoid valves. (B) Illustration of buoyancy control through adjustment of the robot’s soft-shell pressure, enabling underwater lifting. (C) Real-world underwater test showing the robot’s ability to move upward 40 cm in 5 s. (D) Demonstration of omnidirectional underwater crawling via coordinated control of the robot’s 6 arms, achieving a 70 cm crawl in 55 s along a fixed direction. (E) Continuous upward transportation of multiple objects underwater, highlighting the robot’s potential for automated underwater debris collection. By remotely evacuating air from the shell to induce sinking, and subsequently refilling it to restore buoyancy, the robot can effectively grasp and transport objects.

Fig. 6A presents a schematic diagram of the OUT-Robot design, featuring a gripper with variable stiffness. The robot’s shell consists of an external silicone soft layer and an internal skeleton (for details on the fabrication of the OUT-Robot, see the Supplementary Materials, Fig. S12). The control of the water pump and all solenoid valves is managed by the circuit board inside the robot (Fig. S13). The robot’s drive system is enclosed within the skeleton and is waterproof (Figs. S14 and S15). Four water inlets and outlets are used to control the robot’s swimming. Crucially, the propulsion mechanism operates independently of the grasping state. After the gripper secures an object via SMP shape locking, the specific solenoid valves isolate the arms from the hydraulic circuit (Fig. S14). This allows the water pump to be fully dedicated to generating thrust, ensuring that the robot’s mobility is not compromised by the holding action. The robot’s buoyancy is controlled to achieve underwater lifting by adjusting the pressure of the robot’s soft shell (Fig. 6B and Movie S4). In a real underwater environment, the robot achieved an upward movement of 40 cm within 5 s (Fig. 6C and Movie S4). Referring to the previous work [15], the robot can realize underwater movement through the coordinated control of its 6 arms. For example, when any 5 arms of the robot are bent to the maximum state simultaneously, the pressure in these arms is released, and the robot moves in the direction indicated by the unbent arm. Since the arms are distributed in different directions, the robot can achieve omnidirectional crawling underwater by controlling the bending and release of different arms. The robot crawled 70 cm in 55 s along a fixed direction, demonstrating its ability to crawl underwater (Fig. 6D and Movie S4). Next, the robot was used to carry out a continuous upward transportation test of multiple objects underwater (Fig. 6E and Movie S5). First, the robot’s shell was depressurized to increase its density and sink in the water. When the robot swims above the object, the grasping mode was turned on to complete the grasping. Subsequently, the robot shell was inflated with air to increase its buoyancy, causing it to float and drive the grasped object upward. Next, the same task was performed 3 times in a row to complete the upward transportation of multiple objects. We further analyzed the energy efficiency of this operational cycle (Fig. S17). The system utilizes a “Pulse-Actuation, Zero-Retention” strategy: The grasping phase consumes a total of ≈75 J (57.6 W peak for 1.3 s) to soften the SMP, while the subsequent shape locking holds the object with zero energy. Similarly, buoyancy activation requires only ≈10 J (≈5 W for 2 s) to inject air, with the subsequent ascent driven passively by hydrostatic forces (0 W). This profile demonstrates a substantial reduction in the total energy budget compared to continuous actuation systems. The robot has shown great potential for application in addressing submerged waste on the seafloor. In the future, it can autonomously and continuously perform the task of grasping and transporting debris upward without human intervention. This is not only expected to replace traditional manual cleaning methods and reduce labor costs and safety risks, but more importantly, it provides a new and efficient solution for protecting the marine environment.

## Discussion

In this paper, we have developed an underwater gripper and the OUT-Robot platform that addresses key challenges in adaptive manipulation, specifically overcoming the thermal hysteresis bottleneck of soft actuators. While the gripper features good biomimicry, its core scientific contribution lies in its novel thermodynamic architecture. Critically, this design validates the effectiveness of environmental coupling in soft robot design. Unlike terrestrial SMP actuators that struggle with cooling, our system turns the underwater environment from a thermal burden into an active component (heat sink) of the control loop. This is realized through a specialized trilayer thermal interface (an inner silicone thermal diffuser, a nickel–chromium heating layer, and an outer transient thermal barrier). This structure not only protects the SMP from localized overheating but also optimizes the heat flux dynamics, enabling the gripper to soften in 1.3 s and rigidify in just 0.8 s—substantially faster than previously reported rates. Furthermore, this architecture ensures that shape locking is rapid and passive. Unlike traditional pressure-driven systems that require continuous pressure to maintain a grasp, our scheme eliminates the need for a continuous energy supply to maintain the rigid state. This “zero-energy” shape-locking capability establishes a foundation for energy-efficient, long-duration underwater manipulation missions. Moreover, this scalable thermodynamic strategy offers rapid response potentials for soft microgrippers [46–48] and energy-efficient shape locking for continuum robots [49].

Additionally, we have established a “Soft-Rigid Hybrid” manipulation strategy that enhances adaptive grasping by strategically decoupling mechanical compliance from structural rigidity. By integrating variable stiffness with multimodal actuation (modes II, IV, VI, and suction), the gripper addresses the classic trade-off between adaptability and load capacity. During the approach phase, the initial soft state allows the arms and suckers to conform perfectly to complex, irregular surface textures, thereby maximizing the effective contact area for suction. Subsequently, the rapid rigidification “freezes” this conformal grasp, locking the shape to support heavy loads without requiring continuous high-pressure actuation. This hybrid strategy substantially amplifies the load-bearing capacity beyond what is achievable by pneumatic force or suction alone, effectively bridging the gap between soft and rigid manipulators. While the current system demonstrates robust performance in cluttered scenes, we acknowledge that real-world underwater missions require active object identification. Future work will focus on integrating underwater computer vision to identify object features and autonomously select the optimal grasping mode (e.g., activating specific arms based on object geometry). Additionally, tactile sensing will be incorporated into the suckers to verify adhesion quality in turbid environments where visual feedback is compromised, closing the loop for fully autonomous manipulation.

Finally, the OUT-Robot establishes a novel “Pick-and-Float” operational paradigm. Unlike conventional underwater vehicles that rely on continuous propeller propulsion, this system effectively separates the localized, energy-intensive grasping task (powered by arm actuation) from the global, energy-efficient transport task (powered by variable buoyancy). This mechanism minimizes acoustic noise and hydraulic turbulence, creating a stealthy operation cycle particularly suited for interacting with sensitive marine ecosystems or performing long-duration ecological cleanup missions where minimizing disturbance is critical. Furthermore, the design opens the potential for a scalable deployment model: Lightweight, autonomous OUT-Robots could be deployed in swarms to perform distributed collection tasks, substantially expanding the operational envelope beyond what singleremotely operated vehicles (ROVs) can achieve. All validation experiments were conducted in a laboratory pool at a depth of 2 m to demonstrate preliminary feasibility. Crucially, while the hydraulic manipulator’s bending angle and load capacity remain unaffected by hydrostatic pressure due to water incompressibility, the compressible air-based buoyancy system limits the current prototype to nearshore, shallow-water applications (0 to 50 m). The target operational environment for nearshore missions typically ranges from 0 to 30 °C, where the system maintains robust functionality due to the substantial thermal differential between the ambient water and the SMP’s glass transition temperature (≈66 °C). This specific operational niche positions the OUT-Robot as a silent, gentle complement to heavy-duty deep-sea ROVs, specifically targeting ecologically sensitive shallow waters.

## Data Availability

All data needed to evaluate the conclusions in the paper are present in the paper and/or the Supplementary Materials.
