# Supplementary material for: Octopus-Inspired Underwater Gripper with Rapid Stiffness Tuning and Robot Enabling Upward Transport
Source: Cyborg Bionic Syst. 2026 Mar 31;7:0528. doi: 10.34133/cbsystems.0528 (PMC13036364; doi:10.34133/cbsystems.0528)
Supplement: Supplementary 1 — Figs. S1 to S17 Movies S1 to S5 [file cbsystems.0528.f1.zip › Revised Supplementary Information.docx]

Supplementary Information for

**Octopus-Inspired Underwater Gripper with Rapid Stiffness Tuning and Robot Enabling Upward Transport**

Mingxin Wu^1,2^, Yurong Liu^3^, Jiaxi Wu^2^, Waqar Hussain Afridi^2^, Xingwen Zheng^4^, Chen Wang^2^, Guangming Xie^2,5^*

^1^National Center for International Joint Research of Micro-Nano Molding Technology, School of Mechanics and Safety Engineering, Zhengzhou University, Zhengzhou, 450001, China

^2^State Key Laboratory for Turbulence and Complex Systems, Intelligent Biomimetic Design Lab, School of Advanced Manufacturing and Robotics, Peking University, Beijing, 100871, China

^3^ Department of Mechanical Engineering, National University of Singapore, Singapore, 117575, Singapore

^4^Institute of Cyber-Systems and Control, Department of Control Science and Engineering, Zhejiang University, Hangzhou 310027, China

^5^Institute of Ocean Research, Peking University, Beijing 100871, China

* Corresponding author: Guangming Xie ([xiegming@pku.edu.cn](mailto:xiegming@pku.edu.cn))

Supplementary Text

Fig. S1. Schematic diagram of the manufacturing process for the stiffness-variable system (A), sucker (B), and actuator (C)

Fig. S2. Manufacturing process of the arm

Fig. S3. Schematic diagram of the sucker driven by three pressures

Fig. S4. Softening performance of variable stiffness system

Fig. S5. Adaptive bending ability of the “soft SMP” arm

Fig. S6. Arm performance characterization system

Fig. S7. Ventral membrane manufacturing steps

Fig. S8. Schematic diagram of the underwater gripper

Fig. S9. Adaptive deformation of the softened gripper in suction mode

Fig. S10. Underwater gripper performance characterization system

Fig. S11. Comparison table of underwater gripper’s grasping modes

Fig. S12. Schematic diagram of mold design and casting of the soft shell of the octopus-like robot

Fig. S13. Internal structure and printed circuit board design of the octopus-like robot

Fig. S14. Design of the drive system for controlling the arm

Fig. S15. Waterproof sealing of the octopus-like robot

Fig. S16. Material behavior

Fig. S17. S17 Energy consumption profile of the underwater gripper system across four operational phases

Movie S1. Positive and negative pressure drive of all suckers

Movie S2. Underwater performance of the SMP-equipped arm

Movie S3. Rapid adjustment of stiffness to achieve continuous grasping in stacked scenes

Movie S4. Underwater movement of the OUT-Robot

Movie S5. Continuous upward transportation of multiple objects underwater

**
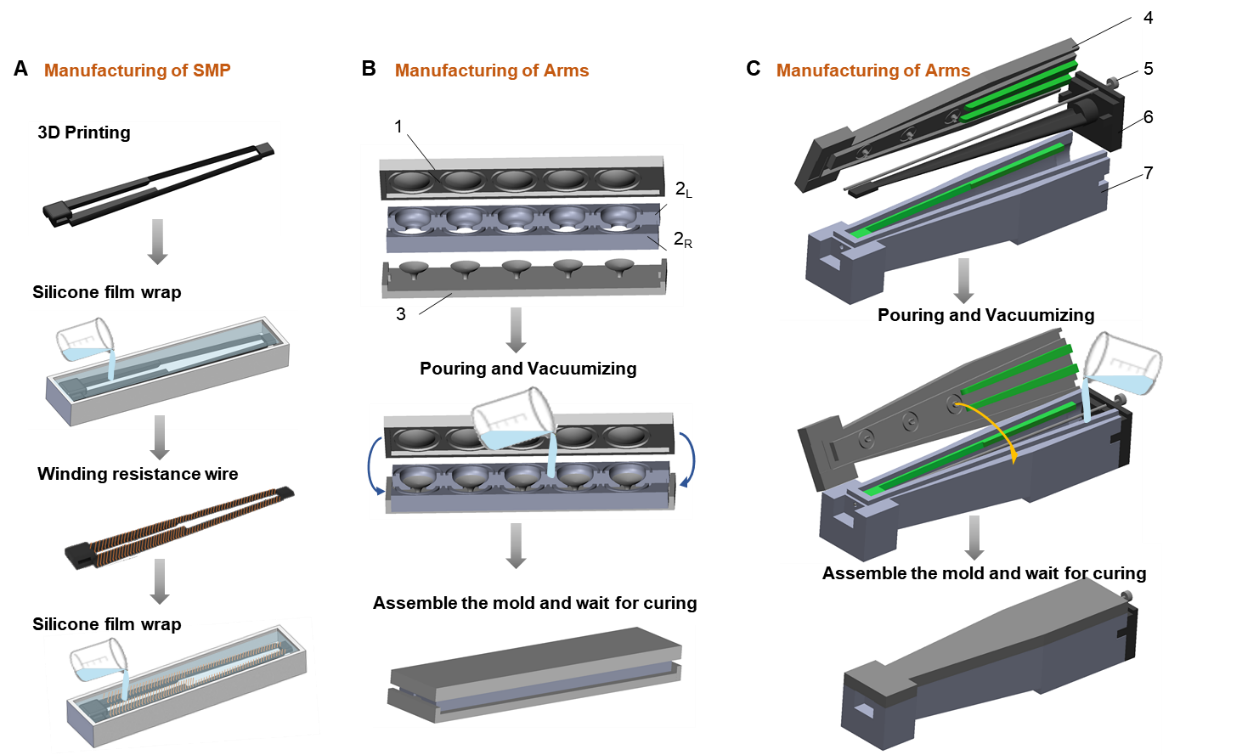
**

**Figure S1.** **Schematic diagram of the manufacturing process for the stiffness-variable system (A), sucker (B), and actuator (C)**

As shown in Figure S1A, the fabrication process of the Shape Memory Polymer (SMP) is presented. Following the design of the SMP model using SolidWorks, it was manufactured using an FDM 3D printer (Polarbear S5). The printing material used was polylactic acid (PLA), with a print speed of 10 mm/s, a nozzle diameter of 0.4 mm, a layer height of 0.3 mm, and a printing temperature of 220 °C. After the completion of printing, the SMP was immersed in silicone (Ecoflex 00-50) and subjected to a vacuum drying process for 30 minutes in a vacuum dryer. Subsequently, the SMP was suspended to allow the silicone coating on its surface to fully cure. Thereafter, resistance wires (composed of nichrome alloy, with a diameter of 0.2 mm) were continuously wrapped around the SMP at 2 mm intervals. Subsequently, the SMP with the resistance wires wrapped around it was immersed again in the silicone solution and subjected to another 30-minute vacuum drying process. Following this, the SMP was suspended once more to allow the silicone coating to fully cure. The silicone serves to stabilize the position of the resistance wires, preventing any movement, and isolates them from the external environment.

The manufacturing process for the suckers and actuator, as illustrated in Figures S1B and S1C, is described below. The modular molds were designed in SolidWorks and sliced using Materialise Magics software, followed by printing with a Stereolithography (SLA) 3D printer. The printer (SLA550) and the printing material (photosensitive resin) were purchased from Zhongrui Technology Co., Ltd. After printing, a casting process was employed to manufacture the suckers and actuator. As shown in Figure S1B, the mold for manufacturing the suckers consists of four parts: 1, 2_R_, 2_L_, and 3. Part 3 is used to form the internal space of the suckers, while parts 1, 2_R_, and 2_L_ determine the external contour of the suckers. Initially, parts 2_R_, 2_L_, and 3 were assembled. Then, silicone (Dragon Skin 20, Smooth-On Inc., PA, degassed in a vacuum chamber for 10 minutes) was poured into the mold, and part 1 was assembled. The silicone was allowed to cure at room temperature for 6 hours. As depicted in Figure S1C, the mold for manufacturing the actuator comprises four parts: 4, 5, 6, and 7. The channel formed by part 5 was used to connect all suckers in series to create suction. Part 6 was used to form the internal chamber of the arm. First, parts 5, 6, and 7 were assembled, and silicone was poured into the mold and degassed in a vacuum chamber for 10 minutes. Subsequently, mold 4 was placed on top, and the silicone was allowed to cure at room temperature for six hours.

**
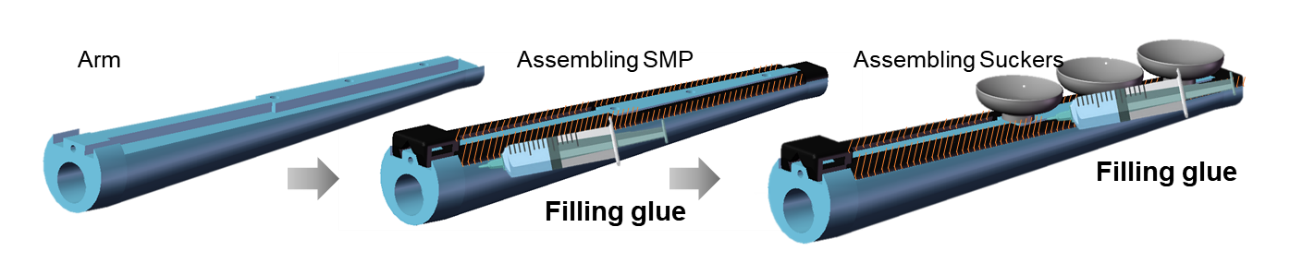
**

**Figure S2.** **Manufacturing process of the arm**

As shown in Figure S2, the variable stiffness system, sucker, and actuator are assembled using an adhesive (Sil-Poxy, supplied by Smooth-On Inc., PA) to form a complete arm. During the manufacturing of the actuator, space has been reserved for embedding the SMP (Shape Memory Polymer). Next, the SMP is inserted into this space, and silicone adhesive is applied to the interface between the actuator and the SMP for bonding. After waiting for 30 minutes, the sucker is then adhered to the actuator in sequence, completing the manufacture of a single complete arm.

**
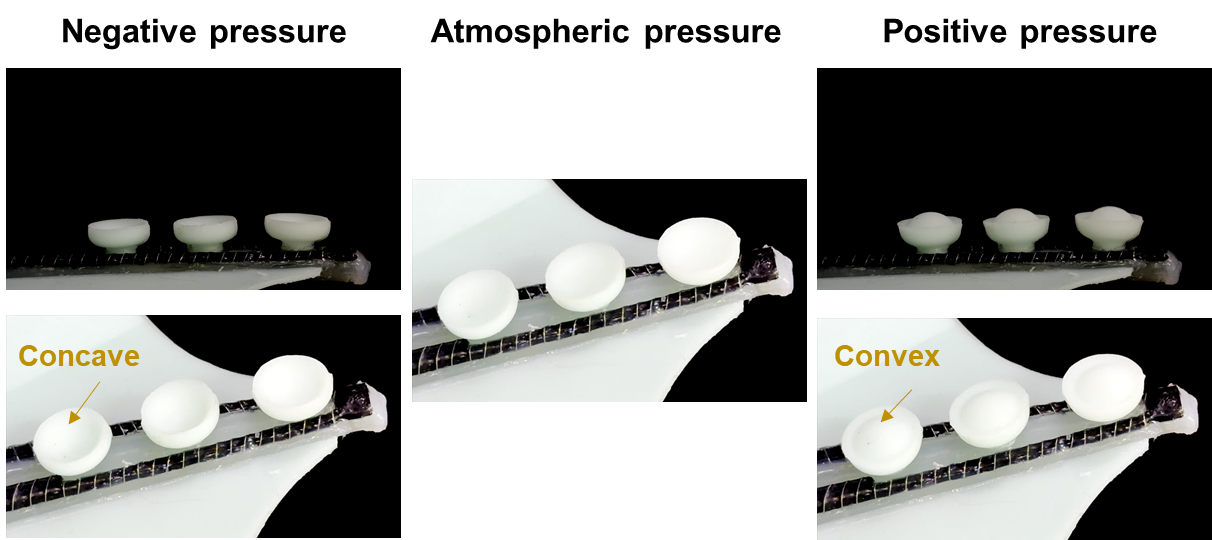
**

**Figure S3.** **Schematic diagram of the sucker driven by three pressures**

Under atmospheric pressure, the sucker is initially in a concave state, providing a pre-adsorption force and adhering to the surface of the object in advance when grasping. In a negative pressure state, the suction force of the sucker is enhanced. On one hand, it can firmly attach to the object's surface to achieve grasping, and on the other hand, it can assist other grasping modes. When positive pressure is applied, the sucker assumes a convex state, allowing it to actively push against and control the grasping and releasing of the object. The conversion between positive and negative pressure for all suckers is controlled in a unified serial manner.

**
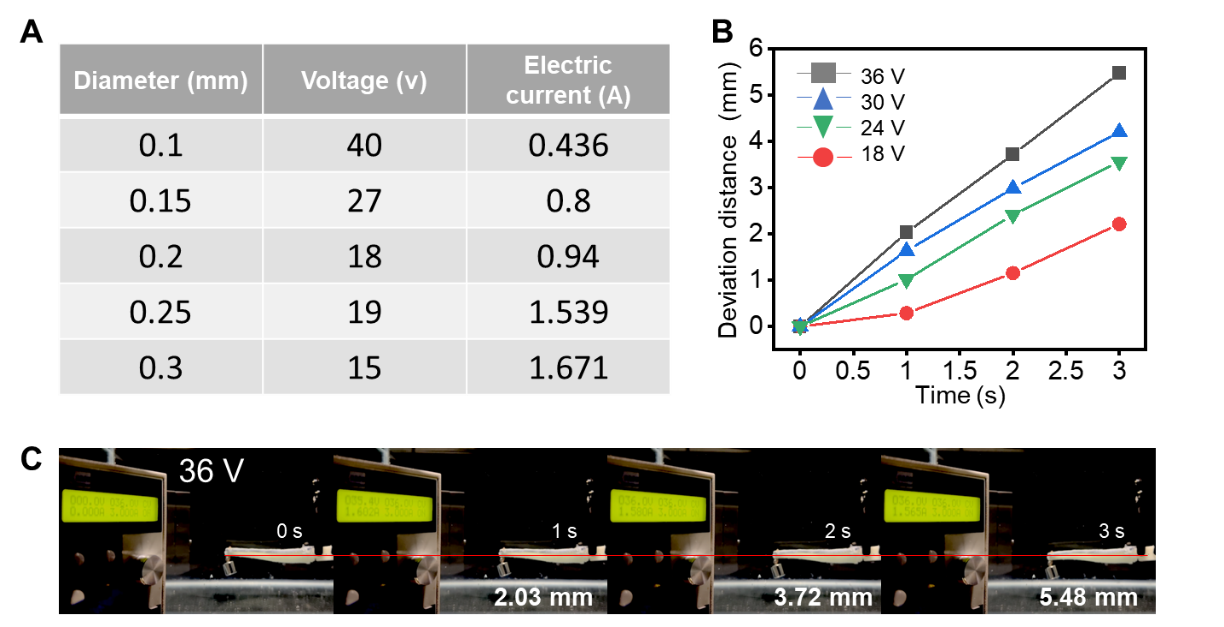
**

**Figure S4.** **Softening performance of variable stiffness system. (A)** Comparison of heating performance of resistance wires with different diameters. **(B)** Comparison of heating performance of resistance wire with a diameter of 0.2 mm under different voltages. **(C)** Softening response of resistance wire under 36 V voltage drive.

To analyze the softening performance of nichrome wires of different diameters on a variable stiffness system, the heating performance of nichrome wires with diameters of 0.1 mm, 0.15 mm, 0.2 mm, 0.25 mm, and 0.3 mm was tested as shown in Figure S4A. As the diameter of the nichrome wire increases, its stiffness also increases accordingly, which in turn affects the bending performance of the arm. Therefore, a quantitative analysis was conducted to choose a nichrome wire with a smaller diameter and a reduced required driving voltage. With a 20 g weight suspended at the end of the arm, the voltage was adjusted, and the minimum voltage and corresponding current required for each nichrome wire diameter to heat up and cause the arm to bend were recorded. Through comparison, it was found that when the arm's (or more specifically, the selected nichrome wire's role in the arm system's) relevant diameter was 0.2 mm, the minimum driving voltage required was 18 V, with a corresponding current of 0.94 A. When the nichrome wire diameters were 0.1 mm and 0.15 mm, or 0.25 mm and 0.3 mm, the driving currents were higher, and the thicker nichrome wires could potentially affect the stiffness of the arm. Ultimately, a 0.2 mm diameter nichrome wire was chosen to wrap around the shape memory material to fabricate the variable stiffness system.

Figure S4B illustrates the response speed of the variable stiffness system using a 0.2 mm diameter nichrome wire at 18 V, 24 V, 30 V, and 36 V, respectively. With a 20 g weight suspended at the end of the arm, the voltage was adjusted, and the displacement of the arm's end over time was recorded. As the voltage increases, the Joule heating rate of the nichrome wire increases, allowing the shape memory strip to heat up and passively bend more rapidly, resulting in displacement at the arm's end. As shown in Figure S4B, the relationship between time and displacement is approximately linear. Figure S4C presents the displacement of the arm's end at 36 V, where a 2.03 mm displacement occurs in the first second, indicating that the shape memory strip begins to soften at that point. Over time, the displacements in the second and third seconds are 3.72 mm and 5.48 mm, respectively. In the variable stiffness system of the gripper, we use 0.2 mm diameter nichrome wire and drive it at a constant 36V to achieve stiffness adjustment.

We derive an empirical, physics-informed model that links the material hardness *H* to the electrical drive current I, heating-wire diameter d, and heating time t by coupling Joule heating, the transient thermal response, and the temperature-dependent mechanical softening of PLA as measured by DMA. The model development is based on the following assumptions: the nichrome wire length is fixed and its DC resistance scales inversely with cross-sectional area (*R ∝ 1/d^2*); Joule heating power is given by $P = I^{2}R$ and a constant fraction of P is transferred to the sampled PLA; the thermal response of the assembly is approximated as a first-order system characterized by a single time constant τ; and the PLA mechanical softening is governed by the storage modulus *E′(T)* obtained from DMA and can be mapped to hardness by a linear scaling factor.

**Electrical-to-thermal coupling**

For a fixed wire length, the DC resistance is approximated as $R\approx\frac{k_{R}}{d^{2}}$, where k_R_ is a lumped geometric/material constant. The input power delivered to the system therefore scales as

$$P_{\mathrm{in}}\propto\frac{I^{2}}{d^{2}}.$$

We introduce a lumped electrothermal coupling constant *A* (units: °C·mm^2/A^2) to relate input power to steady-state temperature rise at the location of interest:

$$\Delta T_{ss}=A\frac{I^{2}}{d^{2}}.$$

**Transient temperature response**

The local temperature *T(t)* is modeled as a first-order response toward the steady-state rise:

$$T(t)=T_{0}+\Delta T(t)=T_{0}+A\frac{I^{2}}{d^{2}}\left( 1-e^{-t/\tau} \right),$$

where *T_0_* is the ambient temperature and τ is the thermal time constant of the wire–coating–polymer assembly.

**Temperature-to-mechanical mapping**

The storage modulus *E′(T)* of PLA exhibits a sharp glass-to-rubber transition around *Tg* (DMA measurement), which we represent with a smooth logistic function:

$$E^{'}(T)=E_{\mathrm{low}}+\frac{E_{\mathrm{high}}-E_{\mathrm{low}}}{1+\exp\left( \frac{T-T_{g}}{\beta} \right)},$$

where *E_high_* and *E_low_* are the glassy and rubbery storage moduli, *T_g_* is the glass transition temperature, and *β* controls transition steepness. Hardness *H* is taken proportional to *E′* via a constant *c*:

$$H(t)=c\cdot E^{'}\left( T(t) \right).$$

**Final combined model**

Substituting the transient temperature into the *E′* expression and scaling to hardness yields the closed-form model used for fitting:

$$H(I,d,t)=c\left[ E_{\mathrm{low}}+\frac{E_{\mathrm{high}}-E_{\mathrm{low}}}{1+\exp\left( \frac{T_{0}+A\frac{T^{2}}{d^{2}}(1-e^{-t/t})-T_{g}}{\beta} \right)} \right].$$

**
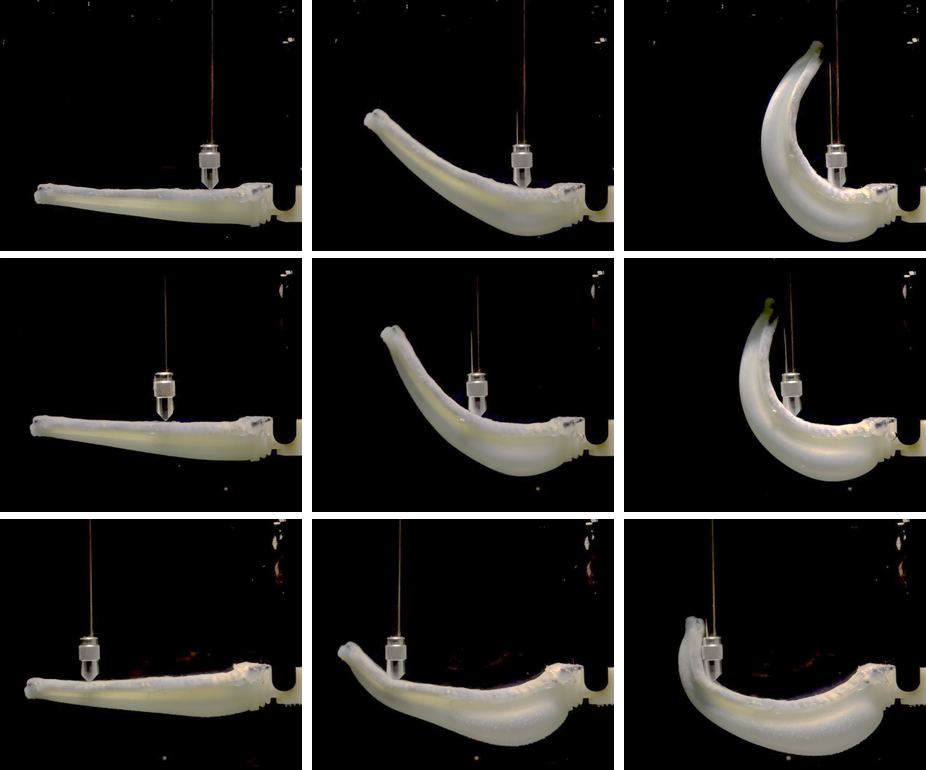
**

**Figure S5.** **Adaptive bending ability of the “soft SMP” arm**

The adaptive bending capability of the "soft SMP" arm was tested in an underwater scenario to determine whether the gripper can achieve adaptive grasping of irregular objects. During the measurement, the resistive wire wrapped around the shape memory material was activated, heating up and causing the arm to soften. Subsequently, the arm was driven by positive pressure, and the bending states of different positions along the arm were measured when encountering obstacles. As shown in Figure S5, the bending states of three positions on the arm were measured when they encountered obstacles. The "soft SMP" arm forms joints at the positions where it is blocked, while the segments closer to the tip of the arm continue to bend. This demonstrates that, after the incorporation of SMP, the arm maintains the high compliance characteristics of a soft robot when softened, allowing it to exhibit adaptive grasping capabilities for irregular objects.

**
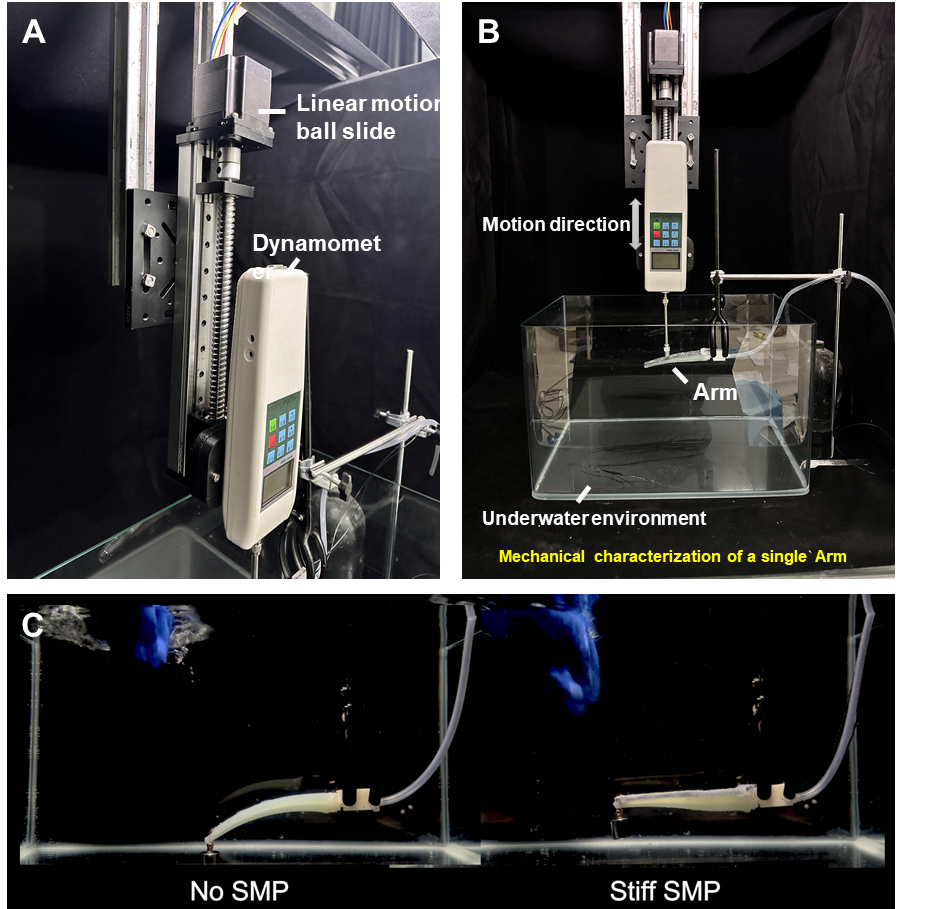

Figure S6.** **Arm performance characterization system**


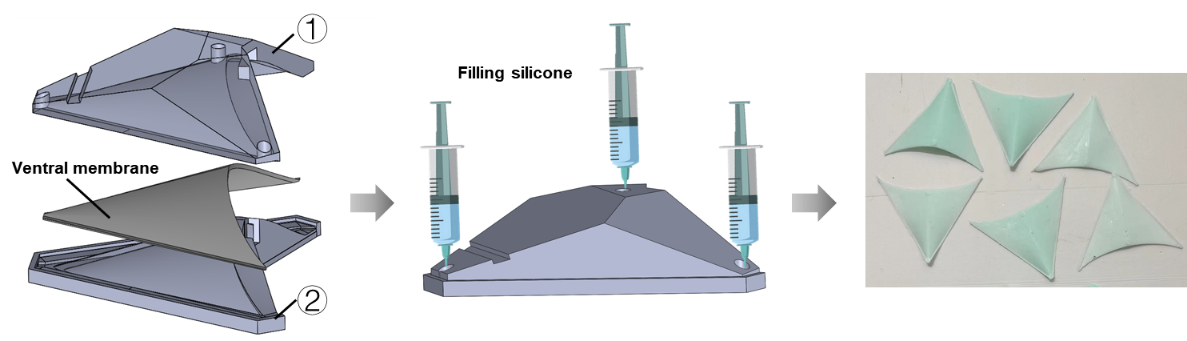


**Figure S7.** **Ventral membrane manufacturing steps**

The manufacturing steps for the ventral membrane involve dividing the mold into two parts: the upper mold ① and the lower mold ②, in order to form a ventral membrane with an arcuate curve. Firstly, assemble and securely clamp the upper and lower molds together. Then, inject silicone (Dragon Skin 20, Smooth-On Inc., PA, which has been degassed in a vacuum chamber for 30 minutes) through the four silicone inlets. Stop the injection when silicone overflows from the inlets, and then seal the inlets. Allow the silicone to cure at room temperature for 4 hours.


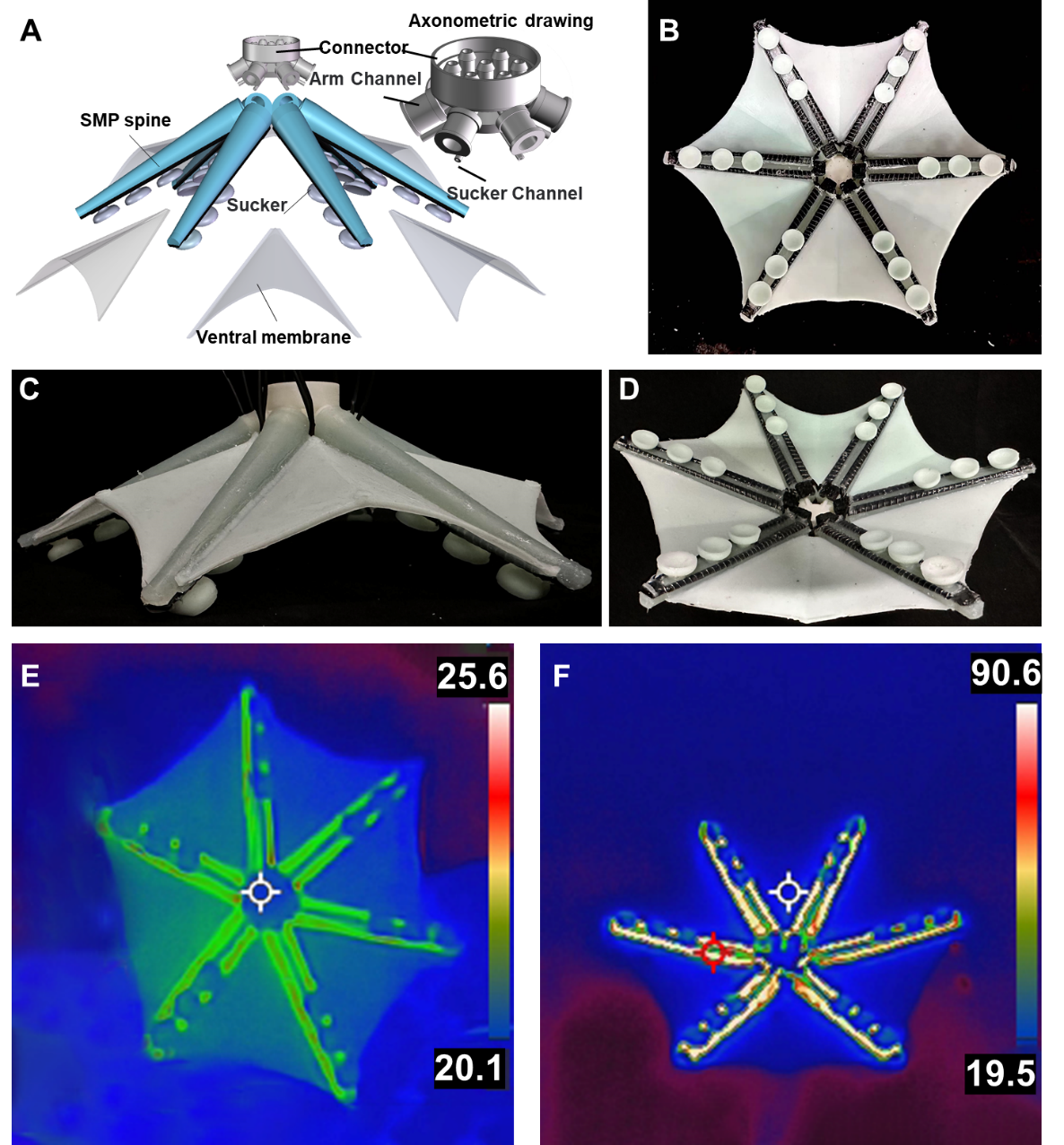


**Figure S8.** **Schematic diagram of the underwater gripper.** (A) The gripper consists of a connector, six arms, and six ventral membranes. (B-D) Diagram of the underwater gripper. (E-F) Thermal imaging of the gripper arm heating up in air

**
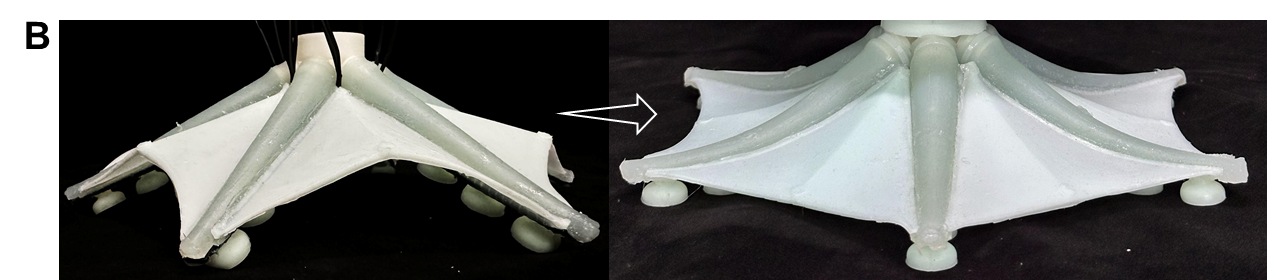
**

**Figure S9.** **Adaptive deformation of the softened gripper in suction mode**

**
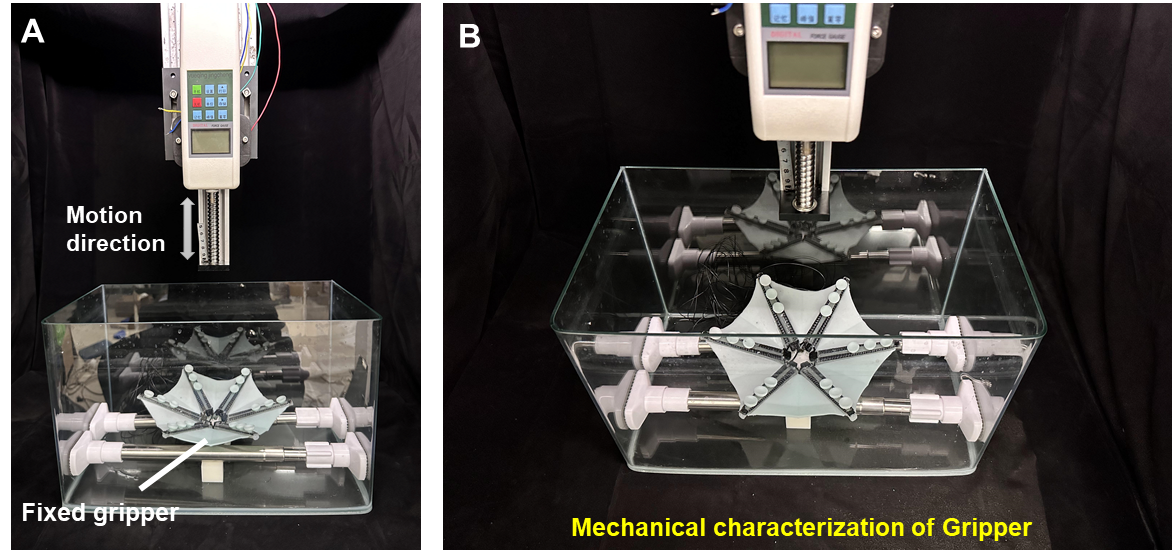
**

**Figure S10.** **Underwater gripper performance characterization system**

The gripper was fixed underwater, and its mechanical performance was characterized using a force measurement platform. For grasping experiments, objects with three different diameters (55 mm, 75 mm, and 95 mm) were used in Mode II and Mode IV, while objects with diameters of 75 mm, 95 mm, and 145 mm were used in Mode VI.

**
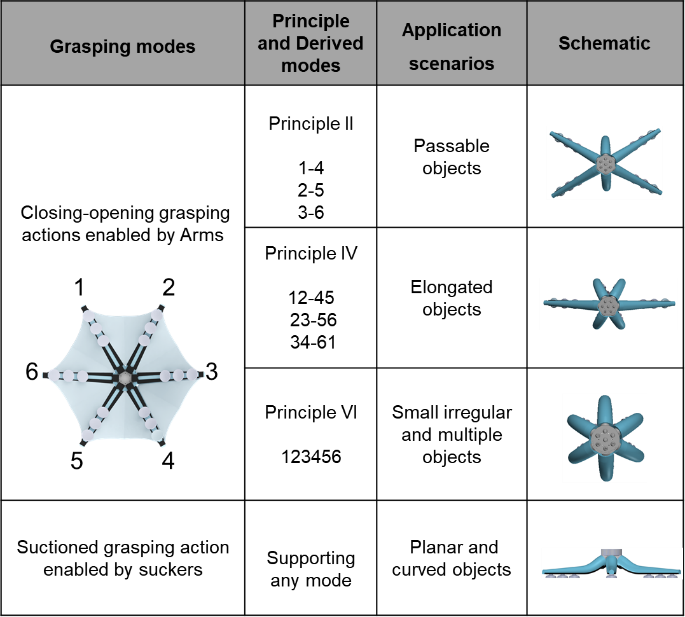
**

**Figure S11.** **Comparison table of underwater gripper’s grasping modes**

Figure S11 illustrates the various grasping modes of the gripper integrated with a variable stiffness system. As shown in Figure 4, the red SMP indicates that the arm is being heated and is in a softened state, allowing for actuated bending and grasping. The black SMP signifies that heating has stopped, and the arm has hardened due to the cooling effect of the external environment, enhancing the stiffness at the tip for improved grasping stability.

The first mode is Mode II, which involves actuating any two symmetrically positioned arms. To initiate this mode, the SMP is first electrically heated to soften. Subsequently, any two symmetrically positioned arms are bent and passed through an object with holes. The power is then turned off, and the arms rapidly harden in the low-temperature underwater environment, completing the stiffness adjustment and enhancing the stability of the grasp.

The second mode is Mode IV, which involves actuating any four symmetrically positioned arms. To activate this mode, the SMP is first electrically heated to soften. Then, any four symmetrically positioned arms are bent to grasp a long, cylindrical object. During this process, the two unactuated arms conform to the grasped object. At this point, the negative pressure suction of the suckers can be activated to assist with grasping. Once the four actuated arms are bent and conform to the grasped object, the power is turned off. The arms rapidly harden in the low-temperature underwater environment, completing the stiffness adjustment and enhancing the stability of the grasp.

The third mode is Mode VI, which involves actuating all six arms. To initiate this mode, the SMP is first electrically heated to soften. Then, all six arms are bent to grasp an object smaller than the gripper's shape. When the arms are bent and conform to the grasped object, the power is turned off. The arms rapidly harden in the low-temperature underwater environment, completing the stiffness adjustment and enhancing the stability of the grasp.

Finally, there is the suction grasping mode. To activate this mode, the SMP is first electrically heated to soften. At this point, all six arms remain unactuated. When the softened arms come into contact with a flat object, they conform to its shape, allowing the suckers to adhere to the surface. The initial concave shape of the arms provides pre-adhesion capability, enabling them to stick to the flat object's surface. The negative pressure in the arms is then activated, enhancing the suction force of the suckers to firmly adhere to the object. Finally, the power is turned off, and the arms harden, completing the object grasping in suction mode.

The control system employs a two-stage driving strategy to achieve deformation control with adjustable stiffness. In the first stage, a constant 36V DC voltage is applied to the shape memory material, heating it to soften and reduce structural stiffness, thereby providing low-impedance conditions for the subsequent pressure-driven stage. Once the material reaches the softened state, the system transitions to the pressure-driven stage, where a constant-flow air pump applies pressure to drive the arm’s bending. The relationship between the bending angle *θ* and applied pressure (P) follows the fitted function:

$$\theta(P)=-0.0013P^{3}+0.2804P^{2}-4.4365P+17.438$$

Upon reaching the target maximum bending angle, the system terminates driving by cutting off power and pressure, allowing the arm to achieve final shape locking under water cooling.

To enhance control precision in future work, an angle sensor will be integrated to collect real-time bending angle data θ. When the angle error satisfies the tolerance condition$\mid\theta-\theta d\mid<\varepsilon$ (where *θ_d_* is the target angle and *ε* is the preset error threshold), the system will cut off power and pressure to terminate driving.

Additionally, to enable more intelligent closed-loop control, tactile sensing functionality will be incorporated into the suckers. When the majority of suckers on the driven finger detect the preset pressure threshold, indicating stable contact, the system will terminate driving using a PI controller-based closed-loop control strategy.

The tactile sensing system uses pressure sensors to measure the contact pressure *Ps* of the suckers in real time. With the target pressure *P_d_* as the reference, the pressure error is defined as $e(t)=P_{d}-P_{s}$. The PI controller adjusts the air pump’s output pressure *P_c_(t)* according to the control law:

$$P_{c}(t)=K_{p}e(t)+K_{i}\int_{0}^{t}e(\tau)d\tau$$

where *K_p_* is the proportional gain and *K_i_* is the integral gain. The controller regulates *P_c_(t)* to drive the actual pressure *P_s_* toward the target pressure *P_d_.* When a predefined proportion (e.g., 80%) of the sucker array detects a pressure error satisfying $\mid e(t)\mid<\delta$*(*where *δ* is the preset pressure error threshold), the system determines that the contact state is stable and terminates driving by cutting off power and pressure, achieving precise shape locking.


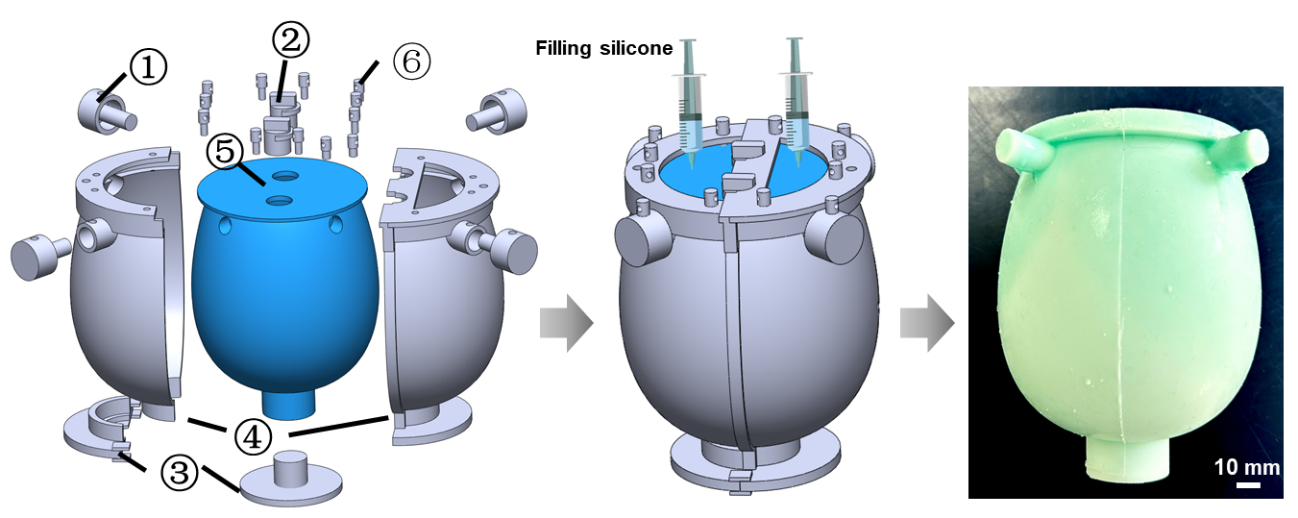


**Figure S12.** **Schematic diagram of mold design and casting of the soft shell of the octopus-like robot**

The soft outer shell is cast integrally using a modular mold system. Module ① is designed to form the inlet and outlet pipes and to fix the position of the core mold, maintaining a consistent gap between the outer mold and the core mold to ensure uniform wall thickness of the soft shell. Module ② serves to secure the core mold ⑤ in place, with an insertion-and-rotation mechanism ensuring the fixed relative position between the core mold and the upper part. Eight locating pins are utilized during the casting process to form bolt holes, which facilitate subsequent bolt tightening and sealing of the robot. Module ③ is responsible for the bottom positioning of the core mold ⑤, while module ④ seals the core mold ⑤, with the gap between it and the core mold ⑤ determining the wall thickness of the soft shell. After assembling all the mold components and ensuring they are pressed and sealed tightly, the mixed silicone is first vacuumed for 10 minutes to remove any air bubbles. Next, a syringe is used to inject the silicone through the locating pin holes until it overflows. Finally, the mold is left to cure at room temperature for 6 hours before being opened. Due to the elasticity of the silicone, the mold can be easily removed to retrieve the soft shell.


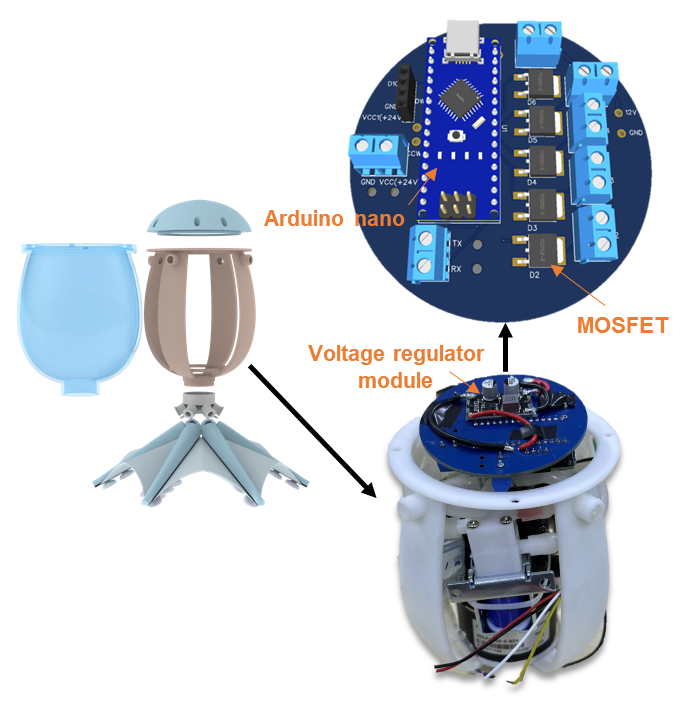


**Figure S13.** **Internal structure and printed circuit board design of the octopus-like robot**


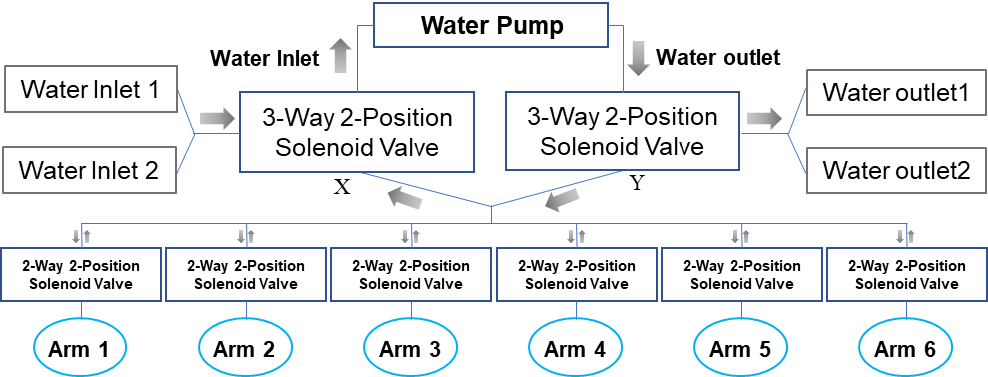


**Figure S14.** **Design of the drive system for controlling the arm**

The internal pump and tubing system of the octopus-like robot is designed as follows: It incorporates a water pump, two three-way solenoid valves, and six two-way solenoid valves. The individual control of each arm is achieved through the opening and closing of the two-way solenoid valves. When Solenoid Valve A is open, the inlet connects to the water pump. When Solenoid Valve A is closed, the X port connects to the water pump. Similarly, when Solenoid Valve B is open, the outlet connects to the water pump, and when Solenoid Valve B is closed, the Y port connects to the water pump. When Solenoid Valve A is open and Solenoid Valve B is closed, the pump draws water from the external environment and supplies it to all arms through the Y port. At this point, by controlling Solenoid Valves 1-6, different arms can be bent to achieve various grasping modes and movement patterns. Conversely, when Solenoid Valve A is closed and Solenoid Valve B is open, the pump draws water from the arms and discharges it out of the robot through the outlet, creating a negative pressure inside the arms. This facilitates the rapid release of the arms after they have been filled with water, accelerating the recovery speed of the bent arms. The control of the water pump and all solenoid valves is implemented through a custom-designed printed circuit board.


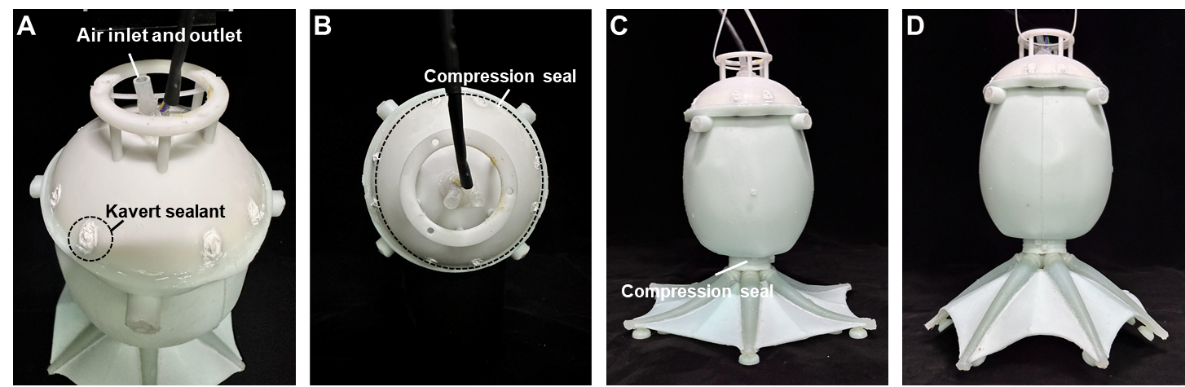


**Figure S15.** **Waterproof sealing of the octopus-like robot.** (A-C) Sealing methods of various joints of the robot. (D) Image of the robot.

Assemble the components and make the octopus-like robot waterproof. As shown in Figure S15 (A-B), the top shell and the silicone soft shell are pressed and sealed by bolts. Silicone is applied to the joints to enhance the sealing effect, and the bolt joints are also sealed with calvert glue. As shown in Figure S15 (C), the contact surface between the soft shell and the variable stiffness gripper is sealed by pressing. As shown in Figure S15 (D), there is an image of the assembled octopus-like robot.


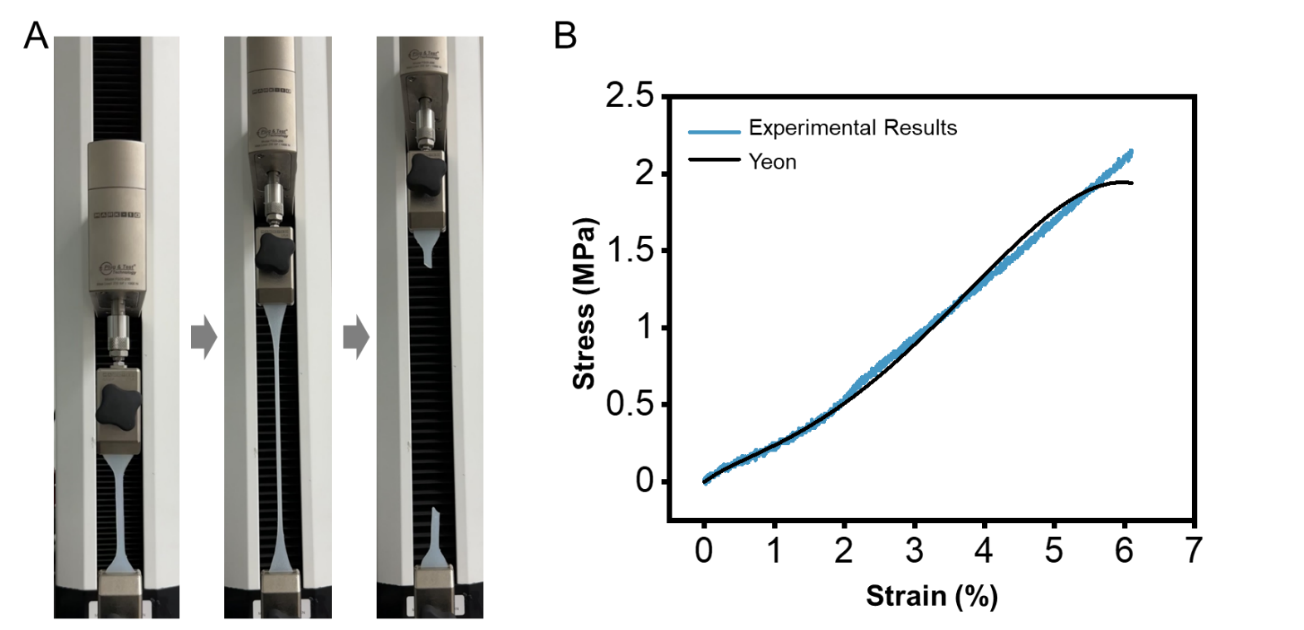


**Fig. S16.** **Material behavior. (A-B)** Stress-strain curve for Dragon Skin 20 as measured in experiments (continuous line) and predicted using a Yeon hyperelastic material model (dashed line).

To characterize the mechanical response of the silicon rubber used to fabricate our Arms, we tested dog bone-shaped samples (ASTM standard) made out of Dragon skin 20 under uniaxial tension, using a single-axis Instron (Mark-10 F105) with a 100 N load cell. The material behavior up to a strain of 6.2 (i.e., until failure) is reported in Fig. S13. We find that the material response was effectively captured by an incompressible Yeon hyperelastic model ^40^, whose strain energy is given by：

$$W=\sum_{i=1}^{N} C_{i0}\left( \bar{I}_{1}-3 \right)^{ⅈ}+\sum_{k=1}^{N} \frac{1}{D_{k}}\left( J-1 \right)^{2k}$$

Where *J* is the volume ratio after deformation to before deformation, for incompressible materials, *J=1.* $I_{1}$ is the first strain tensor invariant.

$$\bar{I}_{1}=J^{-2/3}$$

*C_i0_* and *D_k_* are the material parameters. In our model *N = 3, C_10_ = 0.06066, C_20_ = 0.00224, C_30_ = -1.8532 * 10^-5^ , and D_1_ = D_2_ = D_3_ = 0 .*

**
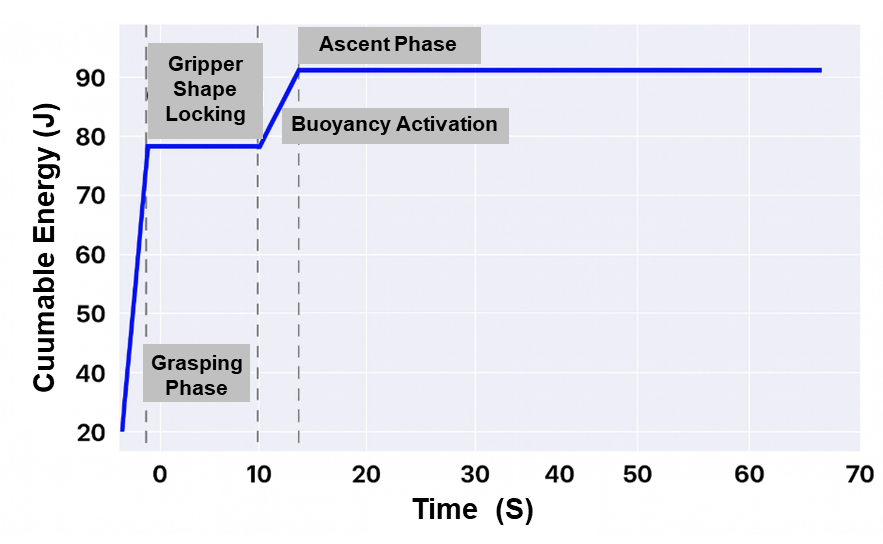
**

**Fig. S17 Energy** **consumption profile of the underwater gripper system across four operational phases**
